# Mononuclear complexes of a tridentate redox-active ligand with sulfonamido groups: structure, properties, and reactivity†
†Electronic supplementary information (ESI) available: Preparative details, Fig. S1–S16, Tables S1–S5 and full crystallographic information for **1** (CCDC 1813042), **1**-Co (CCDC 1813044), **2** (CCDC 1813039), **2**-Co (CCDC 1813041), [M(ibaps)(DMA)_2_] (M = Fe (CCDC 1813040), Ga (CCDC 1813043)). For ESI and crystallographic data in CIF or other electronic format see DOI: 10.1039/c7sc05445a


**DOI:** 10.1039/c7sc05445a

**Published:** 2018-07-02

**Authors:** Sarah A. Cook, Justin A. Bogart, Noam Levi, Andrew C. Weitz, Curtis Moore, Arnold L. Rheingold, Joseph W. Ziller, Michael P. Hendrich, A. S. Borovik

**Affiliations:** a Department of Chemistry , University of California—Irvine , 1102 Natural Sciences II , Irvine , California 92697 , USA . Email: aborovik@uci.edu; b Department of Chemistry , Carnegie Melon University , Pittsburgh , Pennsylvania 15213 , USA; c Department of Chemistry and Biochemistry , University of California—San Diego , San Diego , California 92093 , USA

## Abstract

The design of molecular complexes of earth-abundant first-row transition metals that can catalyze multi-electron C–H bond activation processes is of interest for achieving efficient, low-cost syntheses of target molecules. To overcome the propensity of these metals to perform single-electron processes, redox-active ligands have been utilized to provide additional electron equivalents. Herein, we report the synthesis of a novel redox active ligand, [ibaps]^3–^, which binds to transition metals such as Fe^II^ and Co^II^ in a meridional fashion through the three anionic nitrogen atoms and provides additional coordination sites for other ligands. In this study, the neutral bidentate ligand 2,2′-bipyridine (bpy) was used to complete the coordination spheres of the metal ions and form NEt_4_[M^II^(ibaps)bpy] (M = Fe (**1**) or Co (**1**-Co)) salts. The Fe^II^ salt exhibited rich electrochemical properties and could be chemically oxidized by 1 and 2 equiv. of ferrocenium to form singly and doubly oxidized species, respectively. The reactivity of **1** towards intramolecular C–H bond amination of aryl azides at benzylic and aliphatic carbon centers was explored, and moderate to good yields of the resulting indoline products were obtained.

## Abstract 2

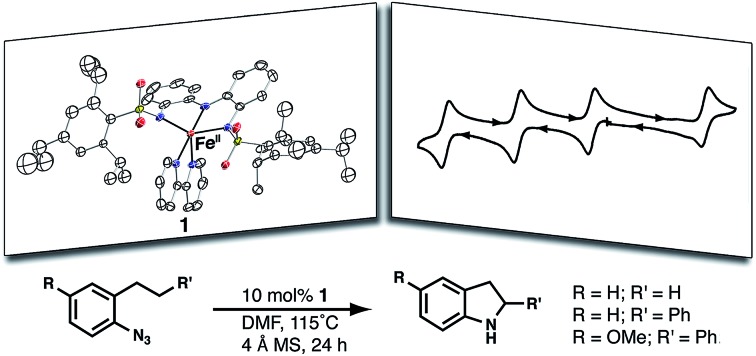
Enhancing the redox properties of Fe^II^ with a bis(sulfonamido)amine pincer ligand leads to catalytic cyclic amination reactivity.

## Introduction

A constant area of interest in synthetic inorganic chemistry is the preparation of highly active molecular catalysts for the functionalization of simple commodity chemicals to form complex fine chemicals.^1^–^9^ While many types of reactions are needed to build complex molecules, one key transformation is the activation of unfunctionalized C–H bonds.^10^–^15^ This reaction is important both in the early stages of molecular synthesis for the conversion of hydrocarbon feedstocks to synthetically useful building blocks and in the late-stage introduction of functional groups in complicated target syntheses. Progress has been made in the field of homogeneous catalysis, with numerous examples of highly active and selective catalysts reported in the literature.^16^ However, the vast majority of these successful catalysts employ expensive second- and third-row transition metals such as palladium and iridium. Renewed efforts have been devoted to replacing these precious metals with earth-abundant first-row transition metal catalysts that are more environmentally and economically beneficial.^8^

The development of first-row transition metal catalysts for C–H bond activation is complicated by the propensity of these metal ions to perform single electron transfer processes. Although two consecutive single electron transfer processes can lead to productive C–H bond activation,^17^ the likely occurrence of unwanted side reactions in these radical pathways is a constant detriment to the yield and selectivity of the reaction as well as to the lifetime of the catalyst. One approach for improving the function of first-row transition metal catalysts in multi-electron transformations is the development of redox-active ligand frameworks that can supply or store electrons during the reaction.^18^–^26^ These ligands contain frontier orbitals that have energies similar to those of the metal centers they are coordinated to, which allows the redox events to be localized on the ligand instead of the metal center. The utility of this approach has been demonstrated by the reactivity of d^0^ metals supported by redox-active ligands in multi-electron transformations, such as the reduction of aryl azide by Zr(iv) and Ta(v) centers to form M–imido complexes.^23^,^24^

**Fig. 1:**
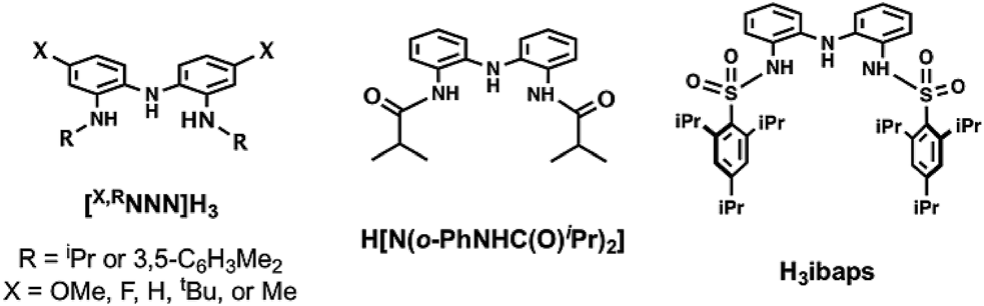
Comparison of the H_3_ibaps ligand used in this work with other reported redox non-innocent [NNN]-pincer ligands.

We have designed a new redox-active ligand that endows Fe and Ga^III^ complexes with rich electrochemical properties. The ligand contains a bis(aminophenyl)aminate ([NNN]^3–^) framework whose redox activity has been previously studied.^23^–^28^ Our ligand differs from those of the previous studies through the functionalization of the redox-active framework with two sulfonamido units (Fig. 1), which we and others have shown can produce metal complexes with diverse structures and functions.^29^–^41^ For this study, we have incorporated sterically encumbering triisopropylbenzene groups to support the formation of a coordinatively unsaturated monomeric Fe^II^ complex. We demonstrated that the metal center remains accessible to endogenous ligands by producing bipyridine adducts whose oxidative properties we have also defined. This Fe^II^ complex of bipyridine exhibited the ability to perform multi-electron processes, including C–H bond amination *via* azide activation.

## Results

### Preparations

**Scheme 1:**
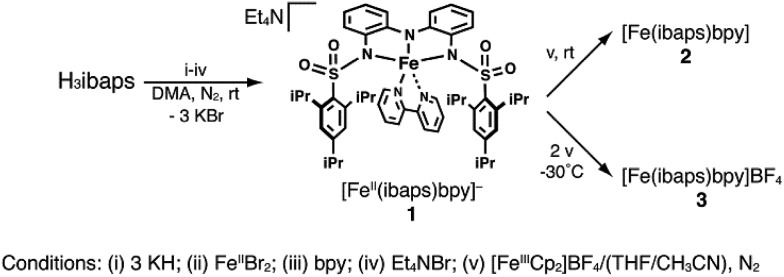
Syntheses of compounds **1–3**.

The ligand precursor *N*,*N*′-(azanediylbis(2,1-phenylene))bis(2,4,6-triisopropyl-benzene-sulfonamide) (H_3_ibaps) was readily prepared from reaction of the triamine precursor bis(aminophenyl)amine^42^ with triisopropylbenzene sulfonylchloride in a yield of 64%. Following deprotonation with three equiv. of KH in dimethylacetamide (DMA), [ibaps]^3–^ coordinated to an Fe^II^ center in a meridional fashion with the phenyl rings of the ligand backbone nearly planar. The coordination sphere of the metal center was completed by the bidentate ligand 2,2′-bipyridine (bpy) to give the potassium salt of the five-coordinate complex [Fe^II^(ibaps)bpy]^–^ (**1**). Without isolation, this salt was metathesized with tetraethylammonium bromide to afford the desired product in a crystalline yield of 73% (Scheme 1). Treating this salt with ferrocenium afforded the one-electron-oxidized analog [Fe(ibaps)bpy] (**2**) in a moderate yield of 64% (see below). In a similar manner, the doubly oxidized species was prepared by treating **1** with 2 equiv. of ferrocenium to produce [Fe(ibaps)bpy]^+^ (**3**) in a yield of 85%.

**Scheme 2:**
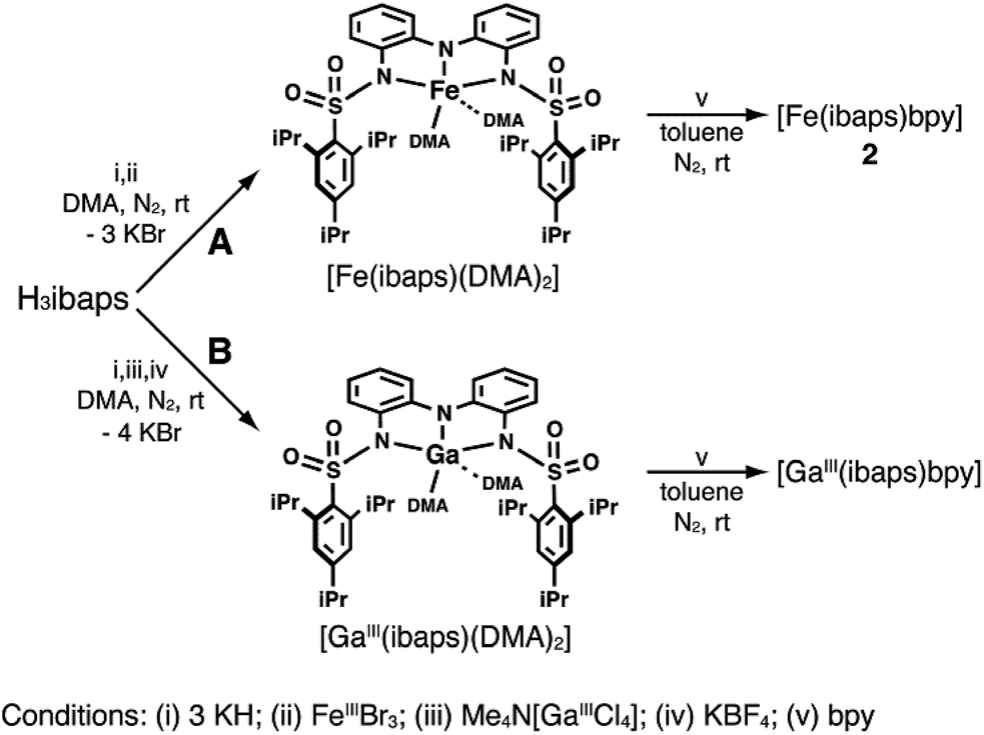
Alternative syntheses of [M(ibaps)bpy] complexes using [M(ibaps)(DMA)_2_] (M = Fe or Ga) as synthons.

An alternative method for the synthesis of **2** was developed in which the iron center was introduced into the complex *via* FeBr_3_ (Scheme 2A). After recrystallization from toluene/pentane, the isolated product had a molecular formula of [Fe(ibaps)(DMA)_2_]. The molecular structure of this product was determined by X-ray diffraction methods and revealed that the two DMA molecules were coordinated to the Fe center through their carbonyl O atoms (Fig. S1, Table S2,† Fe1–O5, 1.90(1) Å and Fe1–O6, 2.001(4) Å) to produce a five-coordinate complex with trigonal bipyramidal geometry. [Fe(ibaps)(DMA)_2_] served as a convenient synthon that could be converted to **2** through treatment with one equiv. of bpy. This method was also used to prepare the corresponding Ga^III^ complex (Scheme 2B): treating [ibaps]^3–^ with (CH_3_)_4_N[GaCl_4_] and one equiv. of KBF_4_ produced [Ga^III^(ibaps)(DMA)_2_], which was isostructural to the Fe complex (Fig. S2, Table S2†). The structure of [Ga^III^(ibaps)(DMA)_2_] in solution was probed by ^1^H NMR spectroscopy in CDCl_3_, and the results are consistent with a five-coordinate complex that retained the coordinated DMA molecules (Fig. S3†). Allowing [Ga^III^(ibaps)(DMA)_2_] to react with one equiv. of bpy produced [Ga^III^(ibaps)bpy] in a 77% isolated yield, and the product was characterized by ^1^H and ^13^C NMR spectroscopies (Fig. S4 and S5†).

### Redox properties

**Fig. 2:**
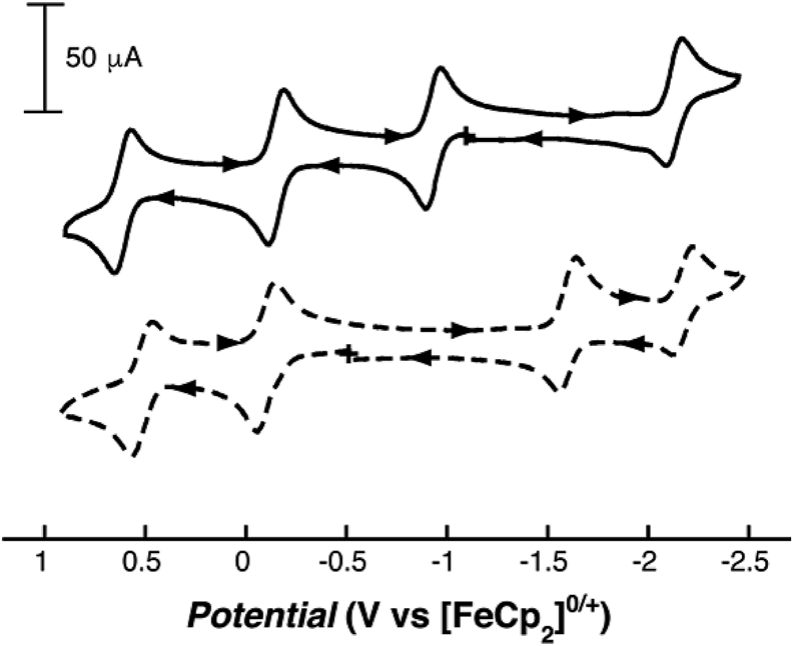
Cyclic voltammograms of **1** (solid) and [Ga^III^(ibaps)bpy] (dashed) at room temperature in CH_3_CN.

The redox behavior of **1** was assessed through solution electrochemistry experiments and referenced to the [FeCp_2_]^+/0^ couple. The cyclic voltammogram (CV) of **1** in CH_3_CN exhibited rich and reversible electrochemical properties, with four reversible events observed (Fig. 2, Table 1). Based on the open circuit potential, these events were assigned as three reversible oxidations and one reversible reduction. The first and second oxidations of **1** occurred at –0.94 V and –0.15 V, respectively. The final oxidative process was observed at a significantly higher potential of 0.60 V. The CVs of **1** measured in THF and DMF possessed features that resemble those found in the CV collected in CH_3_CN but with some noticeable differences (Fig. S6 and S7†). The first and second oxidative events were located at potentials of –0.99 and –0.08 V, respectively, in THF and at –1.10 and –0.15 V in DMF (Table 1). However, the third reversible oxidation observed in CH_3_CN was irreversible in THF and DMF, and additional redox features were introduced that were not observed when the scan was stopped at 0.4 V (Fig. S8 and S9†). This third oxidative feature occurred at nearly the same potential as that in CH_3_CN (*E*_a_(THF) = 0.78 V; *E*_a_(CH_3_CN) = 0.65 V). The cause(s) of the instability of **1** in THF and DMF after the third oxidation is still under investigation.

**Table 1:** Redox potentials of **1** and [Ga^III^(ibaps)bpy]

		**1**			[Ga(ibaps)bpy]		
		MeCN	THF	DMF	MeCN	THF	DMF
Oxidations	*E*_1/2_(1)	–0.94	–0.99	–1.10	—	—	—
	*E*_1/2_(2)	–0.15	–0.08	–0.15	–0.10	–0.052	–0.11
	*E*_1/2_(3)	0.60	—	—	0.52	—	—
Reductions	*E*_1/2_(1)	–2.09	–2.29	–2.18	–1.60	–1.64	–1.79
	*E*_1/2_(2)	—	–2.74	–2.59	–2.18	–2.32	–2.18

In addition to the three oxidative events observed for **1**, the complex exhibited reversible reduction chemistry, with one event observed in CH_3_CN at –2.09 V (Fig. 2). We suggest that this reduction is ligand based; however, the [ibaps]^3–^ ligand is already in its most reduced form, so this reduction is localized on the bpy ligand. Similar potentials have been observed for the reduction of bpy in other transition metal complexes.^43^,^44^ Additional reduction events were observed for **1** in THF and DMF at potentials of –2.74 V (THF) and –2.59 V (DMF) when the potential was scanned more negatively; the currents of those peaks were generally 30% lower than those found for the other couples (Fig. S6 and S7†).

To determine which oxidative event corresponds to the M^II/III^ redox couple, cyclic voltammetry experiments were also performed on the analogous [Ga^III^(ibaps)bpy] complex (Fig. 2). This Ga^III^ complex was chosen because Ga^III^ and Fe^III^ ions have similar ionic radii and coordination chemistries (see above),^45^ but the redox inactivity of the Ga^III^ center allows for the direct investigation of the redox properties associated with the bound [ibaps]^3–^ and bpy ligands. Similar to the cyclic voltammogram observed for **1**, that of [Ga^III^(ibaps)bpy] in CH_3_CN exhibited four reversible one-electron redox processes. However, the observed redox events were assigned as two reversible oxidations and two reversible reductions based on the open circuit potential. The two oxidative events observed at –0.10 and 0.52 V for [Ga^III^(ibaps)bpy] were assigned to the [ibaps]^3–/2–^ and [ibaps]^2–/1–^ couples, respectively. These processes occurred at potentials similar to those of the second and third oxidative events of **1**, which supports the assignment of these features as originating from the [ibaps]^3–^ ligand. In addition, the lack of an oxidative process for [Ga^III^(ibaps)bpy] at a more negative potential suggests that the first oxidation in **1** is metal centered.

The first reductive event observed for [Ga^III^(ibaps)bpy] at –1.60 V, which is assigned to the [bpy]^0/–^ redox couple, occurred at a potential ∼0.5 V less negative than the same event in [M^II^(ibaps)bpy]^–^. This difference in the redox behavior of the bound bpy between the complexes resulted from the differing overall charges of the complexes. For the [Fe^II^(ibaps)bpy]^–^ complex, the first reduction probed an ion with an overall –1 charge, whereas that for the [Ga^III^(ibaps)bpy] complex probed a neutral species. Therefore, in [Ga^III^(ibaps)bpy], the reduction of the bpy ligand should occur at a higher potential, as observed. A second reduction event was observed at –2.18 V for [Ga^III^(ibaps)bpy] and was assigned to the [bpy]^1–/2–^ redox couple. This redox process was not observed for **1** under the same experimental conditions because of the overall charge difference. Note that when the CV of **1** was collected in THF and DMF, a second reduction process (see above) consistent with the [bpy]^1–/2–^ couple was observed (Table 1, Fig. S6 and S7†).

### Molecular structures

**Fig. 3:**
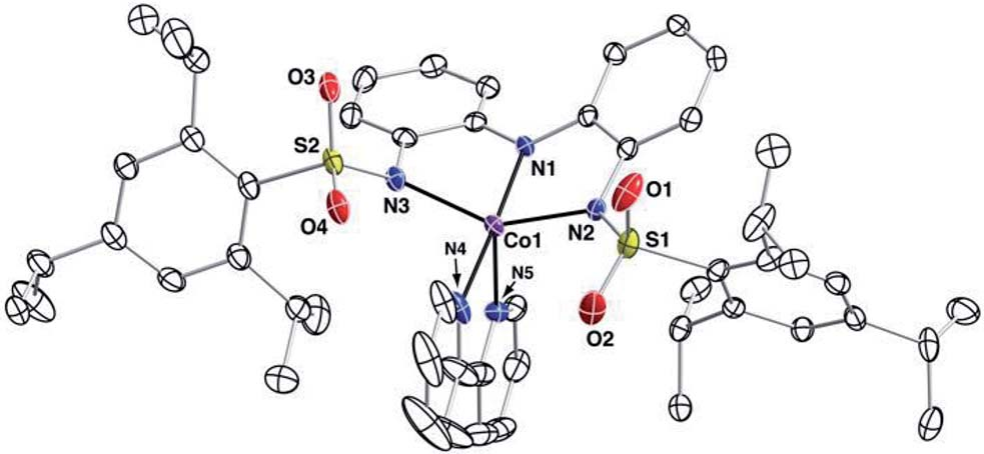
Thermal ellipsoid diagram of **1**-Co. The ellipsoids are drawn at the 50% probability level, and hydrogen atoms are omitted for clarity.

The molecular structure of **1** was determined by XRD methods, which confirmed that the complex is five-coordinate. However, only a low-resolution structure could be determined (Fig. S10†). To gain additional structural insight into the complexes with the [ibaps]^3–^ ligand, we prepared and analyzed the molecular structure of the corresponding [Co^II^(ibaps)bpy]^–^ complex (**1**-Co), which gave higher resolution data (Fig. 3); **1**-Co was synthesized analogously to **1** except CoBr_2_ was used for metalation. The discussion of the Co^II^ analog is confined to these structural findings, and the full details of this complex will be published elsewhere. An overlay of the molecular structures of **1** and **1**-Co indicated that these two complexes adopt similar solid-state coordination geometries (Fig. S11†).

In both complexes, the structure around the metal center is best described as midway between square pyramidal and trigonal bipyramidal. This assessment is based on the trigonality structural parameter (*τ*_5_), which ranges from *τ*_5_ = 0 for a square pyramidal geometry and *τ*_5_ = 1 for a trigonal bipyramidal geometry: the structure of **1**-Co has a *τ*_5_ value of 0.51 (Table S4†).^46^ One of the N-atom donors of the bpy ligand coordinates almost exactly opposite of the deprotonated amine (N1) in **1**-Co with an N1–Co1–N4 bond angle of 177.2(1)°, which lengthens the Co1–N4 bond distance by 0.055(3) Å relative to the Co1–N5 bond length (2.132(3) *vs.* 2.077(3) Å, respectively). The [ibaps]^3–^ ligand coordinates in a meridional orientation with the backbone aryl rings slightly skewed from planarity, as indicated by a dihedral angle of 33.75°. The sulfonamido oxygen atoms frame the open coordination sites on the M^II^ centers, with the bulky aryl groups of the sulfonamido positioned to surround the bpy ligand.

**Fig. 4:**
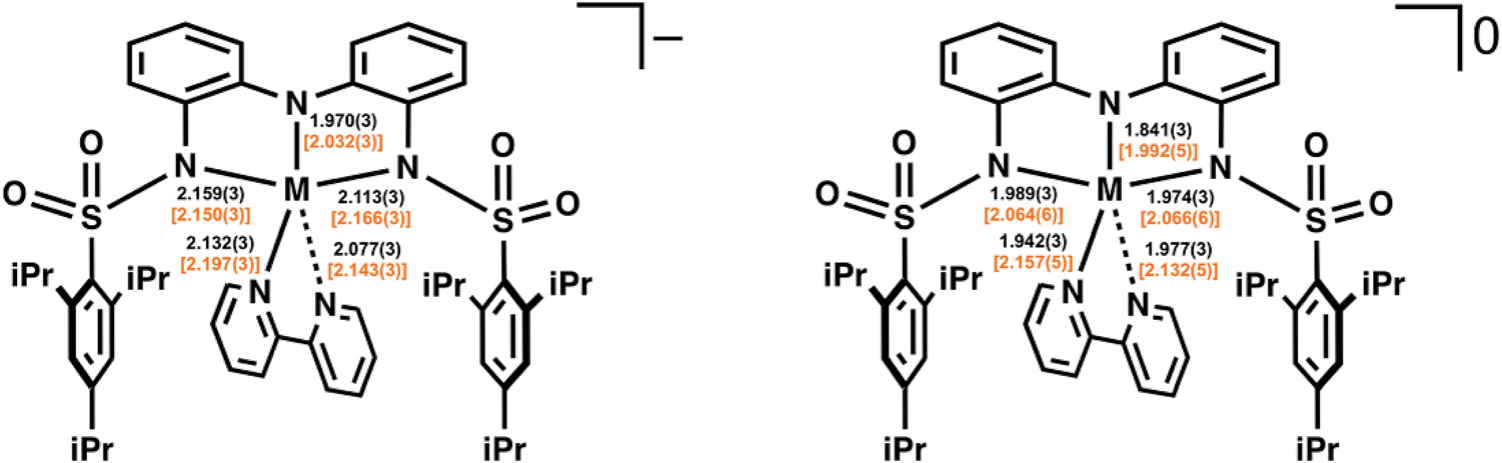
Comparison of the bond lengths (Å) for the M–L bonds in [**1**-M]^–^ (left) and **2**-M (right) (M = Fe, Co). The values for the cobalt structures appear unbracketed in black above those bracketed in orange for the iron structures.

The molecular structure of the one-electron-oxidized complex of **1**-Co (denoted **2**-Co, Fig. S12†) was also obtained. Single crystals of **2**-Co contained two crystallographically independent but virtually identical molecules in the asymmetric unit. Structural data for one of the molecules are provided in Table S4,† and the data for the second molecule of **2**-Co are listed in Table S5.† The [ibaps]^3–^ ligand in **2**-Co is still coordinated in a meridional manner, but statistically significant differences in the metrical parameters are observed around the Co1 center relative to those found in **1**-Co. For instance, a shortening of all the Co1–N bond lengths by 0.10–0.19 Å was observed in **2**-Co. In contrast, only small differences between the two complexes were found in the bond lengths within the aryl backbone of the [ibaps]^3–^ ligand (Fig. 4, Table S4†).

The molecular structure of **2** was obtained, but it was also a low-resolution model (Fig. S13†). Nevertheless, the qualitative changes in the bond lengths between **1** and **2** followed a similar trend as that observed for the Co analogs, albeit with smaller overall differences. For example, the differences in the Fe–N_ibaps_ bond lengths between **1** and **2** range from 0.05–0.10 Å, with smaller changes observed in the Fe–N_bpy_ bond distances (Fig. 4, Table S4†). Note that the trend of smaller changes in the bond lengths between **1** and **2** relative to their Co analogs has also been reported in other systems, suggesting that the Fe–N bond distances may not be very sensitive to oxidation.

### Electronic absorbance spectroscopy

Consistent with our electrochemical studies, the addition of one and two equiv. of ferrocenium into UV-vis-NIR samples at –80 °C in THF generated species with new electronic absorbance spectra (Fig. S14†). The one-electron-oxidized complex **2** contained a band at *λ*_max_ (*ε*_M_) = 640 nm (5646) with a shoulder at 550 nm (Table 2) and a lower energy peak at *λ*_max_ (*ε*_M_) = 1025 nm (4082). The energy and intensity of the NIR peak match those found in complexes with related [NNN]^3–^ diaryl pincer ligands, and the studies of those complexes assigned this peak to the one-electron oxidation of the ligand to the semiquinonate oxidation state.^25^ For example, the one-electron oxidation of [(NNN^cat^)Ta(Cl_3_)] ([NNN^cat^]^3–^ = bis(2-(3,5)-diphenyl-aminato-4-methoxy-phenyl)aminato, Fig. 1) produced [(NNN^sq^)Ta(Cl_3_)], which exhibited an absorbance feature at *λ*_max_ (*ε*_M_) = 1035 nm (4500).^25^ The addition of two equiv. of ferrocenium to **1** produced species **3**, which possessed optical features distinct from those of the other Fe complexes. Prominent bands were found in the visible region at *λ*_max_ (*ε*_M_) = 425 (7340), 550 (6910), 700 (sh) and 757 (14 570) nm with a weaker peak at *λ*_max_ (*ε*_M_) = 1000 (1070) nm. These features are similar to those found for [(NNN^q^)Ta(Cl_2_)NC_6_H_4_-(4-CH_3_)] at *λ*_max_ (*ε*_M_) = 364 (17 200), 466 (11 400), 566 (4600), and 774 (15 000) nm, which were assigned to ligand-localized transitions from the doubly oxidized (NNN^q^) ligand.^25^

**Table 2:** Physical properties of **1–3**

Complex	*λ*_max_ nm (*ε*_M_)	*δ* (mm s^–1^)	Δ*E*_Q_ (mm s^–1^)
**1**	438 (sh)	1.03	3.57
**2**	378 (966), 415 (sh), 550 (sh), 640 (5646), 1025 (4082)	0.69	2.55
**3**	425 (7340), 550 (6910), 700 (sh), 757 (14 570), 1000 (1070)	0.26	1.55

The similarities in the absorbance spectra of **2** and [(NNN^sq^)Ta(Cl_3_)] suggest that the one-electron oxidation of [Fe^II^(ibaps)bpy]^–^ produces a species with an electronic structure that possesses significant ligand radical character (that is, the semiquinonate oxidation state). However, this outcome differs from the results of the CV and XRD experiments, which suggested that the one-electron oxidation was a metal-centered process. Moreover, the absorbance spectrum of **3** bears a strong similarity to that of [(NNN^q^)Ta(Cl_2_)NC_6_H_4_-(4-CH_3_)] in which the diaryl-pincer ligand has been oxidized by two electrons to the quinonate oxidation state. These findings suggest that the ibaps ligand in **3** has also adopted quinonate character, and thus, the two-electron oxidation of [Fe^II^(ibaps)bpy]^–^ resulted in appreciable oxidation of the pincer ligand.

### Mössbauer and EPR spectroscopies

**Fig. 5:**
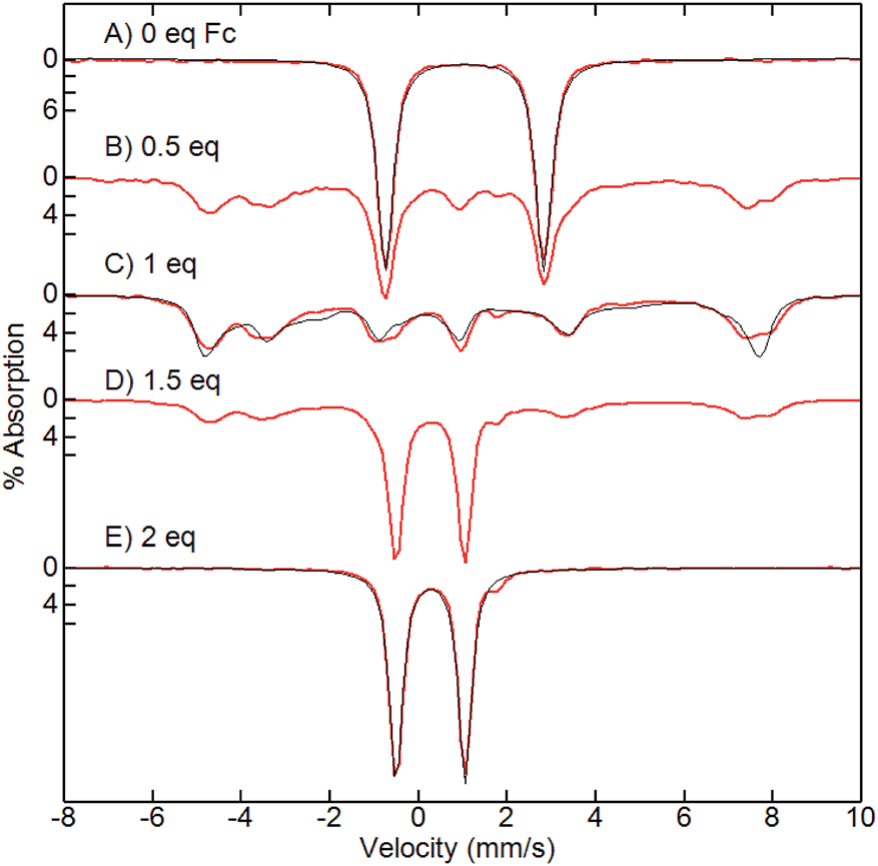
Mössbauer spectra (red traces) and simulations (black traces) of a titration of **1** (A) with ferrocenium (equivalents as shown), recorded at 4.2 K in a magnetic field of 50 mT. For C, the simulation is for an *S* = 2 Fe^II^ exchanged coupled to *S* = 1/2 with Fe^II^ parameters of *D* = 3.5 cm^–1^, *E*/*D* = 0.16, *A* = (–21, –30, –36) MHz, *δ* = 0.69 mm s^–1^, Δ*E*_Q_ = 2.55 mm s^–1^.

To gain insight into the electronic structures of the singly and doubly oxidized species of **1**, Mössbauer and EPR spectra were recorded for a titration series of ^57^Fe-enriched **1** with addition of 0, 0.5, 1, 1.5 and 2 equiv. of ferrocenium. The Mössbauer spectra at 4.2 K in a magnetic field of 50 mT are shown in Fig. 5. Complex **1** had an isomer shift (*δ*) of 1.03 mm s^–1^ and a quadruple splitting (Δ*E*_Q_) of 3.57 mm s^–1^, which are typical of an Fe^II^ center.^47^ The addition of 0.5 equiv. of ferrocenium resulted in a 50% loss of **1** and generation of 50% of **2** which is a paramagnetic species (Fig. 5B). For 1 equiv. of ferrocenium (Fig. 5C), the doublet from **1** vanished and only **2** was present with an *δ* = 0.69 mm s^–1^ and Δ*E*_Q_ = 2.55 mm s^–1^. For an independently prepared powder sample of **2**, the paramagnetic spectrum collapsed into a single doublet with the same parameters. The absence of paramagnetic features was caused by intermolecular spin–spin interactions in powder, which caused high spin relaxation rates.

After addition of 1.5 equiv. of ferrocenium (Fig. 5D), 50% of **2** was lost and 50% of **3** was observed. For 2 equiv. of ferrocenium (Fig. 5E), **3** was observed in nearly 100% conversion with an *δ* = 0.26 mm s^–1^ and Δ*E*_Q_ = 1.55 mm s^–1^. In a magnetic field of 7 T (not shown), the spectrum was indicative of a *S* = 0 diamagnetic species. The Mössbauer parameters are typical of low-spin *S* = 1/2 Fe^III^ centers^47^ and the observation of diamagnetism indicates a spin interaction with a ligand radical.

**Fig. 6:**
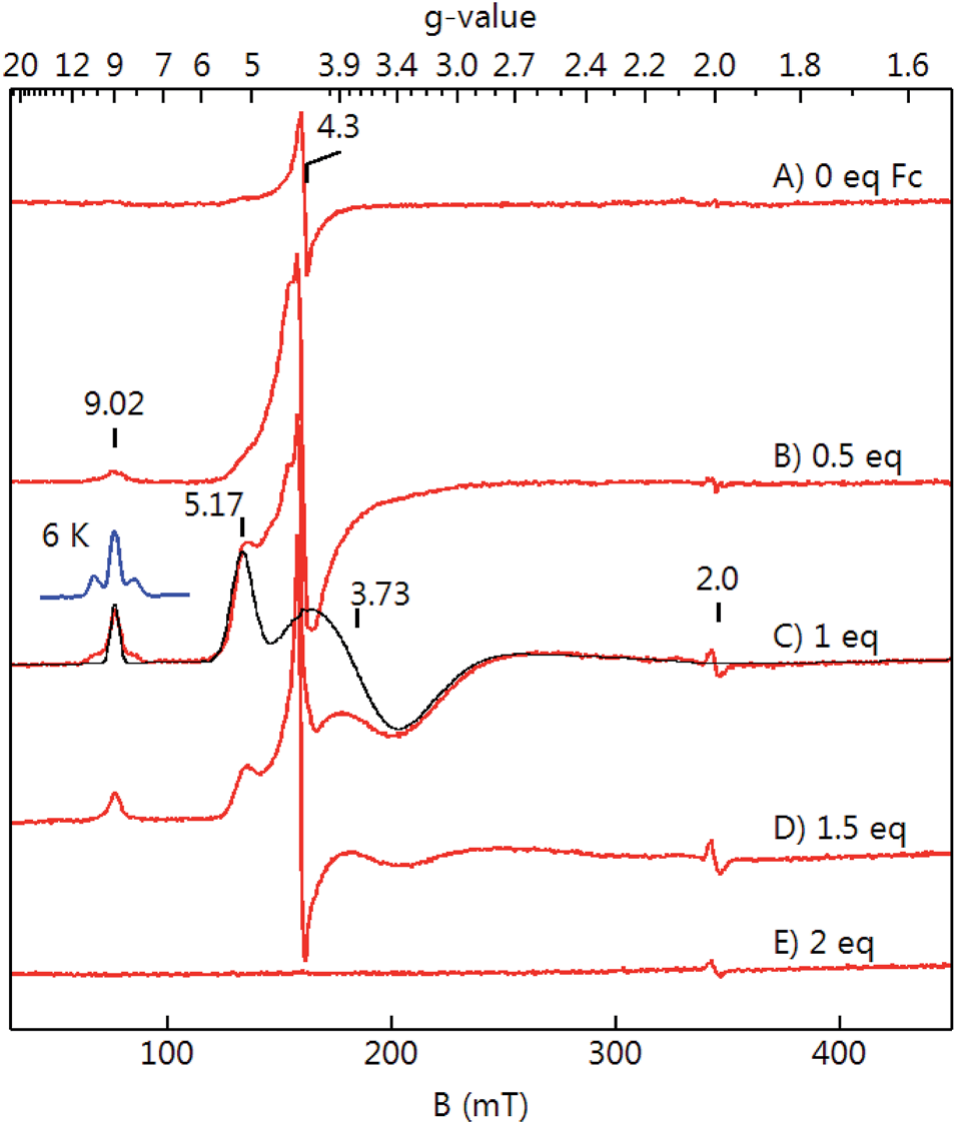
EPR spectra (red traces, 9.646 GHz, 0.2 mW) and simulations (black trace) of a titration of **1** (A) with ferrocenium (equivalents as shown) recorded at 16 K. The simulation is for an *S* = 2 Fe^II^ exchanged coupled to *S* = 1/2 with Fe^II^ parameters of *D* = 3 cm^–1^, *E*/*D* = 0.19.

The EPR spectra of the same titration series (Fig. 6) showed the growth and decay of an *S* = 5/2 species with *g*-values at 9.02, 5.17, and 3.73 that is attributed to **2**. These samples had a minor variable amount of a rhombic *S* = 5/2 species at *g* = 4.3 which amounted to <10% of the iron in the samples. Complex **2** was formed in the greatest amount after the addition of 1 equiv. of ferrocenium and its spin concentration was in agreement with the concentration of the starting complex **1**.

Complex **2** has an isomer shift that is intermediate between Fe^II^ and Fe^III^ centers indicative of partial oxidation of the Fe center owing to a change in the covalency of the Fe–ligand bonds.^47^ Moreover, the quadrupole splitting is inconsistent with high-spin Fe^III^ centers.^47^Fig. 5 and 6 show simulations for an isolated *S* = 2 Fe^II^ center exchange coupled to a *S* = 1/2 radical. For large ferromagnetic *J*, the resulting spin system is *S* = 5/2. The A-tensor is highly anisotropic which is again inconsistent with high-spin Fe^III^. While the simulations approximately agree with experiment, this isolated spin description of the complex is an approximation owing to the metal–ligand bond covalency and presumed spin delocalization. There were additional resonances in the EPR spectrum near *g* = 9 that are not yet understood. These additional resonances were reproducible over multiple independently prepare samples and were best observed at 6 K (Fig. 6C, blue trace). Our analysis of these additional resonances could not be attributed to different species with *S* = 5/2 spin states.

### Reactivity studies


1
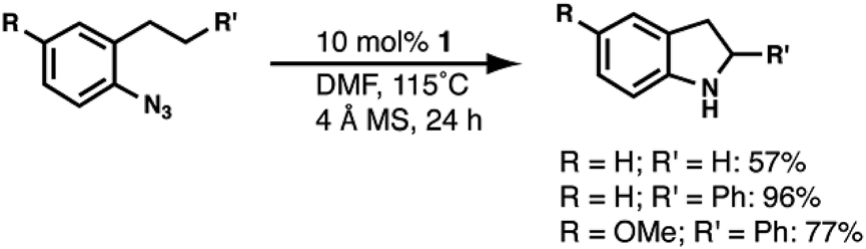
The two-electron chemistry observed for **1** prompted us to explore whether it could serve as a catalyst for the C–H bond amination of aryl azides.^48^–^52^ The substrate 1-azido-2-propylbenzene was used to evaluate the ability of **1** to catalyze the intramolecular amination of non-activated secondary C–H bonds (eqn (1)). The initial screening showed that **1** was unable to catalyze this reaction at room temperature; however, catalysis was observed when the reaction was heated to 115 °C. No reactivity was observed for **1**-Co.

Moderate reactivity at a 10 mol% loading of **1** was observed in 4 : 1 toluene : DMF to afford a 25% yield of 2-methylindoline (Table S6,† entry 1). Loadings of 20 and 50 mol% of the Fe^II^ complex gave only modest increases in the yield of 2-methylindoline (Table S6,† entries 2 and 3). The reaction performed in neat DMF gave the highest yield of the indoline product (57%) (Table S6,† entry 7), whereas the reaction in DMA afforded a lower conversion of indoline (27%) (Table S6,† entry 8), as did the reaction in CF_3_–toluene (17%) (Table S6,† entry 9). This solvent trend suggests that the cyclization pathway towards the indoline product was favorable in more polar solvents. Control reactions with Fe(OAc)_2_/bpy, only H_3_ibaps, and without **1** showed no product formation, and only 1-azido-2-propylbenzene was observed *via* gas chromatography (GC) (Table S6,† entries 10–12).

We also examined a more common substrate that can undergo intramolecular amination of a benzylic C–H bond. Under the optimized conditions found for the reaction with 1-azido-2-propylbenzene, 1-azido-2-phenethylbenzene was converted to the corresponding 2-phenylindoline product in 96% yield (eqn (1)). When 1-azido-4-methoxy-2-phenethylbenzene was used as the substrate, the corresponding indoline was produced in a yield of 77%. However, attempts to activate primary C–H bonds were not successful; no indoline was observed from reaction with 1-azido-2-ethylbenzene.

## Discussion

### Molecular and electronic structures

The [ibaps]^3–^ ligand was designed to bind a single 3d metal ion and facilitate multi-electron redox processes. The key structural aspects of [ibaps]^3–^ are the strong anionic ligand field provided by the three deprotonated N atoms and the appended triisopropylbenzene groups, which provide enough steric bulk to prevent the formation of bimetallic species or [M(ibaps)_2_]^*m*–^ complexes. The meridional coordination geometry of the ligand should render the ligand redox active and provide three coordination sites for additional external ligands. This additional binding was demonstrated by the preparation of [Fe(ibaps)bpy]^*n*^ and [Ga^III^(ibaps)bpy]. Our synthetic and structural studies were consistent with these design criteria.

While the molecular structures of these complexes can be understood, the picture that has emerged for their electronic structures is less clear. The electrochemical properties of [Ga^III^(ibaps)bpy] provide a direct probe of the ligand-based redox events in the absence of the redox-active Fe center. Cyclic voltammetry studies of [Ga^III^(ibaps)bpy] showed two oxidative events corresponding to one- and two-electron oxidation of the [ibaps]^3–^ ligand. The CV of **1** contained an additional oxidative couple from the Fe center that occurred at a lower potential than those found for [ibaps]^3–^, indicating that the first oxidation of **1** is metal centered. Structural studies performed on single crystals of **1** and **2** indicate that this interpretation is reasonable: we observed small but statistically significant changes in the Fe–N bond lengths upon oxidation with minimal changes in the bond lengths within [ibaps]^3–^. However, this interpretation is not consistent with spectroscopic measurements. Mössbauer, optical and EPR spectroscopies suggested that the Fe center in **2** is only partially oxidized and that the [ibaps]^3–^ ligand may also be involved in the redox chemistry. Similar results were found for **3**, in which the Mössbauer parameters indicate a ferric center, whereas the optical spectrum shows peaks that match those expected for the quinonate state of the ibaps ligand. These inconsistent data make it difficult to determine the exact assignments of the oxidation levels of the Fe center and the ibaps ligand in these complexes.

### Reactivity

Complex **1** converts aryl azides to heteroaromatic compounds *via* intramolecular C–H bond activation. Amination at a benzylic position was accomplished using 1-azido-2-phenethylbenzene as a test substrate, and **1** exhibited good reactivity relative to other transition metal complexes. For instance, [(cod)Ir(OMe)]_2_ and [Co_2_(L^iPr^)_2_]^2–^ (L^iPr^ = HN(*o*-PhNHC(O)iPr)_2_) convert the same substrate in yields of 58 and 43%, respectively.^50^,^51^ Complex [Co_2_(L^iPr^)_2_]^2–^ was derived from a similar approach as that of **1** in which a 3d transition metal ion is coupled with a redox-active ligand to perform a two-electron process. Complex **1** also performed intramolecular C–H amination at an aliphatic position, as demonstrated by the production of 2-methylindoline in a moderate yield when 1-azido-2-propylbenzene was used as a substrate. Amination at a secondary carbon center is rare but has been observed in reactions with an Fe^II^(dipyrromethene) complex as well as simple FeBr_2_ salts.^52^,^53^ The FeBr_2_ systems, however, require 20 mol% catalyst loadings and lead to exclusive formation of the indole products as opposed to the indoline products observed in our systems.

The high temperatures needed to perform these intramolecular C–H amination reactions led us to consider the possibility that other species could be catalyzing these reactions. For iron complexes, one potential species is Fe nanoparticles, which can form from traces of water or dioxygen. Dynamic light scattering experiments were done on the reaction mixture, and particles with an average diameter of 5 nm were observed (Fig. S14†). The exact nature of the active catalyst is currently unknown and studies are being performed to determine the role, if any, the nanoparticles have on the amination; however, no reactivity observed under the Fe(OAc)_2_/bpy conditions suggests that the ibaps ligand system affects the catalytic outcome.

## Conclusions

We have developed a trianionic redox-active ligand that readily forms a five-coordinate Fe^II^ complex, **1**, with bpy as an ancillary ligand. Complex **1** can accomplish two-electron chemistry at moderate potentials, as demonstrated by the cyclic voltammetry experiments. Studies on the electronic structures of the oxidized forms of **1** suggest the formation of highly covalent systems in which formal oxidation states cannot be assigned to the Fe centers. For instance, **2** can either be described as [Fe^III^(ibaps)(bpy)] or as [Fe^II^(ibaps˙)bpy] in which the [ibaps]^3–^ ligand has been appreciably oxidized to the semiquinonate form. The ability of **1** to perform two-electron chemistry was further illustrated in reactivity studies toward intramolecular C–H bond activation at benzylic and aliphatic carbon centers, and these studies show the promise of this molecular approach for developing catalysts with earth-abundant metal ions.

## Conflicts of interest

There are no conflicts to declare.

## Supplementary Material

Supplementary information

Crystal structure data
