# Supplementary material for: Mononuclear complexes of a tridentate redox-active ligand with sulfonamido groups: structure, properties, and reactivity
Source: Chem Sci. 2018 Jul 2;9(31):6540–7. doi: 10.1039/c7sc05445a (PMC6115676; doi:10.1039/c7sc05445a)
Supplement: Supplementary file 1 [file SC-009-C7SC05445A-s001.pdf]

Electronic Supplementary Material (ESI) for Chemical Science.
This journal is © The Royal Society of Chemistry 2018

Mononuclear Complexes of a Tridentate Redox-Active Ligand with Sulfonamido Groups: Structure, Properties and Reactivity

Sarah A. Cook,[a] Justin A. Bogart,[a] Noam Levi,[a] Andrew C. Weitz,[b] Curtis Moore,[c] Arnold L. Rheingold,[c] Joseph W. Ziller,[a] Michael P. Hendrich,[b] A. S. Borovik[a]*

[a]Department of Chemistry, University of California—Irvine, 1102 Natural Sciences II, Irvine, California 92697, United States

[b]Department of Chemistry, Carnegie Melon University, Pittsburgh, Pennsylvania 15213, United States, United States

[c]Department of Chemistry and Biochemistry, University of California—San Diego, San Diego, California 92093, United States

Contents

General Methods S2
Physical Methods S2
Ligand Synthesis S5
Complex Syntheses S6
C–H Bond Amination Procedure S9
Sample Preparation S10
Figure S1 S12
Figure S2 S12
Figure S3 S13
Figure S4 S14
Figure S5 S15
Figure S6 S16
Figure S7 S16
Figure S8 S17
Figure S9 S17
Figure S10 S18
Figure S11 S18
Figure S12 S19
Figure S13 S19
Figure S14 S20
Figure S15 S21
Table S1 S22
Table S2 S23
Table S3 S24
Table S4 S25
Table S5 S26
Table S6 S27
Figure S16 S28

Figure S17 S29
Figure S18 S30
Figure S19 S31
Figure S20 S32
Figure S21 S33
References S34

General Methods. The syntheses of metal complexes were completed under a nitrogen atmosphere in a VAC drybox. Solvents were sparged with argon and dried over columns containing Q-5 and molecular sieves. All reagents were purchased from commercial suppliers and used as received unless otherwise noted. Potassium hydride as a 30% suspension in mineral oil was filtered and washed five times each with Et_2O and pentane and dried under vacuum. Ferrocenium tetrafluoroborate ($FcBF_4$) was recrystallized before use. The ligand precursor $HN(o\text{-}PhNH_2)_2$,[1] the Ga starting material $NMe_4[GaCl_4]$,[2] and the aryl azides 1-azido-2-propylbenzene, 1-azido-2-phenethylbenzene, and 1-azido-4-methoxy-2-phenethylbenzene were prepared according to literature procedures.[3]

Physical Methods. Electronic absorption spectra were recorded in a 1-cm cuvette on an 8453 Agilent UV-Vis spectrometer equipped with a Unisoku Unispeks cryostat or in a 1 mm cuvette on a Cary 50 UV-Vis spectrometer. Negative-mode electrospray ionization mass spectra were collected using a Micromass MS Technologies LCT Premier mass spectrometer. Mössbauer spectra were recorded with a Janis Research Super-Varitemp dewar. Isomer shifts are reported relative to Fe metal at 298 K. Fourier transform infrared spectra were collected on a Varian 800 Scimitar Series FTIR spectrometer. ^{1}H NMR and ^{13}C NMR spectra were recorded on a Bruker DRX500 spectrometer and referenced to the residual solvent peak. Elemental analyses were performed on a Perkin-Elmer 2400 CHNS analyzer. Product(s) were detected by gas-chromatography mass spectrometry (GC-MS) in the Mass Spectrometry Facility at the University of California, Irvine. The GC-MS was a Trace MSplus from Thermo Fisher (San Jose,

CA) with a 30-m-long x 0.25 mm i.d. DB-5 column from Agilent JW Scientific (Santa Clara, CA). The mass spectrometer used electron ionization (70 eV) scanning (1/sec) from *m/z* = 50-650. An estimate of the product yield was determined using an HP-6890 gas chromatograph with a flame ionization detector (FID) and a 30 m × 0.32 mm (5% phenyl)-methylpolysiloxane (0.25 μm coating) capillary column (J&W Scientific DB-5) and helium as the carrier gas. DLS measurements were recorded on a Malvern Zetasizer Nano DLS particle size analyzer and data were analyzed by Zetasizer software 7.11.

Electrochemical Measurements. Cyclic voltammetric experiments were conducted using a CHI600C electrochemical analyzer under an N_2 atmosphere with 0.1 M tetrabutylammonium hexafluorophosphate as the supporting electrolyte. A 2.0-mm glassy carbon electrode was used as the working electrode with a silver wire reference electrode and a platinum wire counter electrode. A cobaltocenium/cobaltocene couple (-1.3 V vs. $[FeCp_2]^{+/0}$)[4] was used as an internal reference to monitor the reference electrode (Ag^+/Ag).

X-ray Crystallographic Methods. A Bruker SMART or Bruker Kappa APEX II CCD diffractometer was used to collect data. The APEX2[5] program package was used to determine the unit-cell parameters and for data collection. The raw frame data were processed using SAINT[6] and SADABS[7] to yield the reflection data file. Subsequent calculations were carried out using the SHELXTL[8] program. The analytical scattering factors[9] for neutral atoms were used throughout the analysis. Hydrogen atoms were included using a riding model.

For **[Fe(ibaps)(DMA)$_2$] (DMA = dimethylacetamide).** The structure was solved by dual space methods and refined on F^2 by full-matrix least-squares techniques. There were 1.5 molecules of hexane solvent present per formula unit. One solvent molecule was located about

an inversion center. Several atoms, in particular those of the bound DMA molecules, were disordered and included using multiple components with partial site occupancy factors.

For **[Ga(ibaps)(DMA)$_2$].** The structure was solved by dual space methods and refined on F^2 by full-matrix least-squares techniques. Several atoms were disordered, in particular those of one of the bound DMA molecules and some of the isopropyl groups of the $[ibaps]^{3-}$ ligand, and included using multiple components with partial site occupancy factors. Several high residuals were present in the final difference-Fourier map. It was not possible to determine the nature of the residuals, although it was probable that a toluene solvent molecule was present. The SQUEEZE[10] routine in the PLATON[11] program package was used to account for the electrons in the solvent-accessible voids.

For **1-Co.** The structure was solved by direct methods and refined on F^2 by full-matrix least-squares techniques. Several atoms were disordered, in particular the isopropyl groups of the $[ibaps]^{3-}$ ligand and the ethyl groups of the tetraehtylammonium ion, and included using multiple components with partial site occupancy factors. It was necessary to restrain the distances within the tetraethylammonium ion. There was one half-molecule of diethylether solvent present. The solvent was located about an inversion center and was disordered.

For **1.** The structure was solved by dual space methods and refined on F^2 by full-matrix least-squares techniques. Several atoms were disordered, in particular the atoms of the triisopropylphenyl groups of the $[ibaps]^{3-}$ ligand and the ethyl groups of the tetraehtylammonium ion, and included using multiple components with partial site occupancy factors. Several high residuals were present in the final difference-Fourier map. It was not possible to determine the nature of the residuals, although it was probable that diethylether

solvent was present. The SQUEEZE[10] routine in the PLATON[11] program package was used to account for the electrons in the solvent-accessible voids.

For **2-Co.** The structure was solved by direct methods and refined on F^2 by full-matrix least-squares techniques. There were two molecules of the formula unit and two molecules of tetrahydrofuran solvent present. Carbon atom C(86) of one of the isopropyl groups of the $[ibaps]^{3-}$ ligand was disordered and included using multiple components with partial site occupancy factors.

For **2**. Diffraction pointed to the crystal being cracked/twinned due to desolvation, so PLATON TWINROTMAT was used to deal with some minor, around 4%, contribution from 3 domains. Because of solvent disorder that could not be model, Platon SQUEEZE[10,11] was used to remove the electron density from the lattice due to this solvent contribution. Solvent appeared to be a pentane molecule but was too diffuse to model. Two voids were found with approximately 59 electrons in one and 41 electrons in the other. One isopropyl group of the $[ibaps]^{3-}$ ligand was modeled as a two-component disorder and the thermals parameters were restrained using RIGU to deal with thermal parameter issues.

Ligand Synthesis.

N,N'-(azanediylbis(2,1-phenylene))bis(2,4,6-triisopropylbenzenesulfonamide) (H_3ibaps). The triamine (1.28 g, 6.43 mmol) and pyridine (1.52 g, 19.2 mmol) were dissolved in 50 mL of MeCN in the glove box. Tri-isopropylbenzene sulfonyl chloride (4.08 g, 13.5 mmol) was dissolved in 20 mL of Et_2O and added dropwise to the acetonitrile solution over the course of ten minutes, causing a change from clear orange to heterogeneous dark orange to green. The reaction flask was sealed with a rubber septum, brought out of the glovebox, and fitted with a reflux condensor under a flow of N_2. The reaction was refluxed overnight under N_2 to give a dark red

solution. After checking the reaction for completion by ESI-MS, the mixture was concentrated to dryness under vacuum, redissolved in DCM, and washed with 1M HCl until the aqueous layer was no longer colored. The organic layer was further washed with H_2O and brine, dried over $MgSO_4$, filtered, and concentrated under vacuum to a foam. The ligand precursor was recrystallized from hot iso-octane as light pink crystals in 64% yield (3.0 g). ^1H NMR (500 MHz, d_6-DMSO, ppm): 9.24, (s, 2H, NH), 7.19 (s, 1H, NH), 7.09 (s, 4H), 6.94 (t, 2H), 6.84 (d, 2H), 6.74 (t, 2H), 6.59 (d, 2H), 3.86 (m, 4H), 2.85 (m, 2H), 1.15 (d, 12H), 1.00 (d, 24H). ^{13}C NMR (125 MHz, d_6-DMSO, ppm): 152.0, 150.0, 140.6, 133.4, 128.2, 127.3, 126.0, 123.4, 121.0, 118.9, 33.2, 29.5, 24.6, 23.4. FTIR (Nujol, cm-1, selected bands): 3370 (NH), 3276 (NH), 3213 (NH), 1597 (m), 1314 (s), 1168 (s), 1040 (m), 881 (m), 759 (m). HRMS (ES+): Exact mass calcd (found) for [M + Na]: 754.3688 (754.3680).

Complex Syntheses.

NEt_4[Fe(ibaps)bpy]. A solution of H_3ibaps (0.10 g, 0.14 mmol) in 3 mL of DMA was treated with three equiv of solid KH (16 mg, 0.41 mmol). The reaction was stirred until gas evolution ceased, and then $FeBr_2$ was added. After 1 h, 2,2′-bipyridine and NEt_4Br were added to the reaction, which was stirred for an additional two hours before removing the solvent under vacuum. The crude product was redissolved in 8 mL of THF and filtered through a medium porosity frit to remove three equivalents (49 mg, 0.40 mmol) of insoluble KBr. The product was crystallized from Et_2O vapor diffusion into the THF filtrate to give 0.110 g (73%) of the product as dark green needle crystals. Elemental analysis calcd. (found) for NEt_4[Fe(ibaps)bpy] ($C_{60}H_{82}N_6O_4S_2Fe$): C, 67.27 (67.29); H, 7.72 (8.10); N, 7.84 (7.85) %. FTIR (Nujol, cm^{-1}, selected bands): 1564 (w), 1360 (m), 1231 (s), 1119 (s), 947 (m), 725 (m). λ_{max}, nm (THF, ε, $M^{-1}cm^{-1}$): 438 (sh).

NEt_4[Co(ibaps)bpy]·Et_2O. This complex was prepared according to the same procedure as that for NEt_4[Fe(ibaps)bpy] with H_3ibaps (0.10 g, 0.14 mmol), KH (16 mg, 0.41 mmol), $CoBr_2$ (30 mg, 0.14 mmol), 2,2′-bipyridine (21 mg, 0.14 mmol), and NEt_4Br (29 mg, 0.14 mmol) in 3 mL of DMA. The crude product was redissolved in 6 mL of MeCN and filtered through a medium porosity frit to remove three equivalents (49 mg, 0.40 mmol) of insoluble KBr. The product was crystallized from Et_2O vapor diffusion into the MeCN filtrate to give 0.130 g (86%) of the product as green needle crystals. Elemental analysis calcd. (found) for NEt_4[Co(ibaps)bpy] ($C_{64}H_{92}N_6O_5S_2Co$): C, 66.93 (66.53); H, 8.07 (7.97); N, 7.32 (7.72) %. FTIR (Nujol, cm^{-1}, selected bands): 1597 (m), 1562 (m), 1228 (s), 1118 (s), 1060 (m), 1037 (m), 944 (s), 791 (m), 726 (s).

[Fe^{III}(ibaps)$(DMA)_2$]. To a vial containing a DMA solution of H_3ibaps (0.300 g, 0.411 mmol, 1 equiv) was added solid KH (0.048 g, 1.2 mmol, 3 equiv), and the reaction was allowed to continue for 1 h, after which bubbling ceased and a yellow solution formed. To this solution of the triply deprotonated ligand was added solid $Fe^{III}Br_3$ (0.120, 0.411 mmol, 1 equiv), forming a dark purple solution. After 2 h, the DMA solvent was removed under reduced pressure, and the crude solid was triturated with Et_2O. The compound was extracted from the KBr byproduct with 10 mL of Et_2O and filtered through a medium porosity fritted filter. The filtrate was dried and recrystallized via vapor diffusion of pentane (10 mL) into a toluene (2 mL) solution, which formed large purple X-ray quality crystals after 2 d. Yield: 0.20 g (51%). Anal. Calcd. for Fe(ibaps)$(DMA)_2$, $C_{50}H_{72}N_5O_6S_2Fe$: C, 62.61; H, 7.57; N, 7.30. Found: C, 62.03; H, 7.51; N, 7.10.

[Ga^{III}(ibaps)$(DMA)_2$]. To a solution of the triply deprotonated ligand prepared as above was added solid NMe_4[$GaCl_4$] (0.114 g, 0.411 mmol, 1 equiv), forming a light pink solution. After 3 h, solid KBF_4 (0.051, 0.440, 1 equiv) was added to the reaction mixture, which was stirred for an additional 2 h. The DMA solvent was removed under reduced pressure, and the crude off-white solid was triturated with Et_2O. The compound was extracted from the KCl and NMe_4BF_4

byproducts with 15 mL of Et_2O and filtered through a medium porosity fritted filter. The filtrate was dried and recrystallized via vapor diffusion of pentane (15 mL) into a toluene (3 mL) solution, which formed large colorless X-ray quality crystals after 2 d. Yield: 0.17 g (43%). Anal. Calcd. for Ga(ibaps)$(DMA)_2$•0.5tol, $C_{53.5}H_{76}N_5O_6S_2Ga$: C, 63.06; H, 7.52; N, 6.87. Found: C, 63.09; H, 7.66; N, 6.47. ^1H NMR (500 MHz, $CDCl_3$, ppm): 7.61 (d, J = 7.75 Hz, 2H), 7.08 (s, 4H), 6.74–6.70 (m, 4H), 6.41 (t, J = 7.45 Hz, 2H), 4.61 (sep, J = 6.80 Hz, 4H), 3.23 (br s, 6H), 3.17 (br s, 6H), 2.85 (sep, J = 6.90 Hz, 2H), 2.26 (br s, 6H), 1.22 (d, J = 6.9 Hz, 12H) 1.14 (d, 6.80 Hz, 24H).

[Fe(ibaps)bpy]. *Method A.* To a THF solution of $[NEt_4]$[Fe(ibaps)bpy] (0.10 g, 0.093 mmol, 1 equiv) was added an MeCN solution of $FcBF_4$ (0.025 g, 0.093 mmol, 1 equiv), and the reaction was stirred at room temperature for 2 h. The volatiles were removed under reduced pressure, and the crude solid was washed with pentane. [Fe(ibaps)bpy] was extracted using THF, filtered through a medium porosity fritted filter, and recrystallized via diffusion of pentane into the THF filtrate. Yield: 0.056 g (64%). *Method B.* To a toluene solution of [Fe(ibaps)$(DMA)_2$] (0.096, 0.10 mmol, 1 equiv) was added a toluene solution of 2,2′-bipyridine (0.016 g, 0.10 mmol, 1 equiv), and the solution was allowed to sit at room temperature for 2 h. The microcrystalline solid that precipitated was collected on a medium porosity fritted filter and dried under reduced pressure. Yield: 0.086 g (91%). Anal. Calcd. for Fe(ibaps)bpy•DMA, $C_{56}H_{71}N_6O_5S_2Fe$: C, 65.42; H, 6.96; N, 8.17. Found: C, 64.93; H, 6.91; N, 7.82. λmax, nm (THF, ε, $M^{-1}cm^{-1}$): 378 (9676), 415 (sh), 550 (sh), 640 (5646), 1025 (4082).

[Ga(ibaps)bpy]. This complex was prepared according to *Method B* for Fe(ibaps)bpy but with [Ga(ibaps)$(DMA)_2$]. Yield: 0.073 g (77%). Anal. Calcd. for Ga(ibaps)bpy•DMA, $C_{56}H_{71}N_6O_5S_2Ga$: C, 64.55; H, 6.87; N, 8.06. Found: C, 64.82; H, 6.84; N, 7.86. ^1H NMR (500 MHz, $CDCl_3$, ppm): 9.04 (d, J = 5.00 Hz, 2H), 8.29 (d, J = 8.50 Hz, 2H), 7.99 (t, J = 7.50, 2H), 7.64–7.60 (m, 4H), 6.95 (s, 4H), 6.80 (t, J = 8.00 Hz, 2H), 6.76 (d, J = 7.50 Hz, 2H), 6.41 (t, J = 7.50 Hz, 2H), 4.18 (sep, J = 7.00

Hz, 4H), 2.78 (sep, J = 7.00 Hz, 2H), 1.16 (d, J = 7.00 Hz, 12H), 0.94 (d, J = 7.00 Hz, 24H). ^{13}C NMR (125.8 MHz, 298K, $CDCl_3$, ppm): 151.1, 149.6, 148.6, 147.4, 142.4, 138.8, 136.5, 134.6, 126.5, 123.4, 122.0, 120.6, 117.4, 117.1, 112.7, 34.0, 28.9, 24.7, 23.7.

[Co(ibaps)bpy]. This complex was prepared according to *Method A* for [Fe(ibaps)bpy] but with [NEt_4][Co(ibaps)bpy]. Yield: 0.052 g (60%). Anal. Calcd. for Co(ibaps)(bpy)•0.5THF, $C_{54}H_{66}N_5O_{4.5}S_2Co$: C, 66.17; H, 6.79; N, 7.14. Found: C, 65.64; H, 6.73; N, 6.94.

[Fe(ibaps)bpy]BF_4. To a THF solution of NEt_4[Fe(ibaps)bpy] (0.30 g, 0.279 mmol, 1 equiv) precooled to –35 ºC for 1 h was added an MeCN solution of $FcBF_4$ that was precooled to –35 ºC for 1h (0.153 g, 0.561, 2 equiv), and the reaction was stirred for 5 min every 15 min over the course of 2 h while the temperature was maintained at –35 ºC. The solution was filtered through a precooled medium porosity fritted filter. The filtrate was layered with Et_2O and allowed to sit at –35 ºC for 4 days, after which X-ray quality crystals precipitated. These crystals were collected and dried. Yield: 0.273 g (95%). λ_{max}, nm (THF, ε, $M^{-1}cm^{-1}$): = 425 (7340), 550 (6910), 700 (sh), 757 (14,570), 1000 (1070).

Procedure for the C–H Bond Amination of Aryl Azides. To a DMF solution of **1** (0.0102 g, 0.0095 mmol, 0.10 equiv) was added a DMF solution of aryl azide (0.095 mmol, 1 equiv), and the mixture was diluted to 3 mL with DMF. From this stock mixture, 1 mL was removed and added to a pressure tube equipped with a stir bar and molecular sieves. The pressure tube was sealed, heated to 115 ºC, and allowed to react for 24 h, after which the reaction mixture was removed from the heat. For GC analysis, the reaction mixture was analyzed directly by diluting an aliquot with DCM. For ^{1}H NMR analysis, 100 µL of a 100 mM DCM stock solution of 1,3,5-trimethoxybenzene was added to the reaction as an internal standard before removing the volatiles under reduced pressure. The products were redissolved in ~600 µL of $CDCl_3$ and

filtered. The conversions to indoline were determined by comparing the integration of the singlet for the aryl protons of 1,3,5-trimethoxybenzene at 6.09 ppm to that of the triplet resonance of the indoline product at ~5.0 ppm.

For the reaction of **1** with 1-azido-2-phenethylbenzene: the organic products were isolated using preparative TLC methods with 10% ethyl acetate/hexanes as eluent. The spot corresponding to product (R_f = 0.59) was scraped off and added to a glass funnel plugged with glass wool and washed with DCM. Volatiles were removed under reduced pressure leading to a 41% isolated yield of indoline product.

For the reaction of **1** with 1-azido-4-methoxy-2-phenethylbenzene: the organic products were isolated using preparative TLC methods with 20% ethyl acetate/hexanes as eluent. The spot corresponding to product (R_f = 0.49) was scraped off and added to a glass funnel plugged with glass wool and washed with DCM. Volatiles were removed under reduced pressure leading to a 55% isolated yield of a mixture of indoline and indole products, which could not be further resolved.

Sample Preparation

For Titration Experiments of 1 with [FeCp$_2$]BF$_4$. A 10 mM stock solution of 57-Fe enriched **1** was prepared in THF and 1 mL was added to each of 5 vials and cooled to –35 ºC. A 200 mM stock solution of [FeCp$_2$]BF$_4$ was prepared in MeCN and cooled to –35 ºC. To each vial containing 1 mL of the 10 mM stock solution of 57-Fe enriched **1** was added varying amounts of the [FeCp$_2$]BF$_4$ stock solution (0 µL for 0 equiv; 25 µL for 0.5 equiv; 50 µL for 1 equiv; 75 µL for 1.5 equiv, 100 µL for 2 equiv) and cold MeCN (100 µL for 0 equiv; 75 µL for 0.5 equiv; 50 µL for 1 equiv; 25 µL for 1.5 equiv; 0 µL for 2 equiv) and the vials were allowed to sit at –35 ºC for 2 h. Each were filtered while cold into precooled vials. For Mössbauer, 500 µL of each sample was

diluted with 500 μL of precooled Et_2O and added to a solution Mössbauer cup and frozen in liquid nitrogen. For EPR, 100 μL of each sample was diluted with precooled mixtures of 400 μL THF/500 μL of Et_2O. ~300 μL of each diluted sample was added to an EPR tube and frozen in liquid nitrogen.

For solid-state Mössbauer Studies. A powder of the compound to be analyzed was added to a solid-state Mössbauer cup such that the mass of naturally-abundant ^{57}Fe (2.119%) amounted to ~2 mg. The sample was either capped with a screw-on lid or covered with ~3 drops of Nujol to create a Nujol mull and frozen in liquid N_2 for transportation.

For Dynamic Light Scattering (DLS) Experiments. In a drybox (Ar), (NEt_4)[Fe(II)ibaps(bpy)] (3.4 mg, 0.00317 mmol, 0.1 equiv.) and 1 mL of dry DMF were added into a pressure tube. 1-azido-2-propylbenzene (1 equiv) was added to the solution, and the tube was sealed and taken out of the box. The reaction was stirred at 115-120 °C for 24 h and then allowed to cool to rt. The tube was opened in a drybox, and the reaction mixture was diluted with an additional 2 mL of DMF and transferred to an air-tight 1 cm quartz cuvette and sealed. The sealed cuvette was taken out of the drybox for measurements.

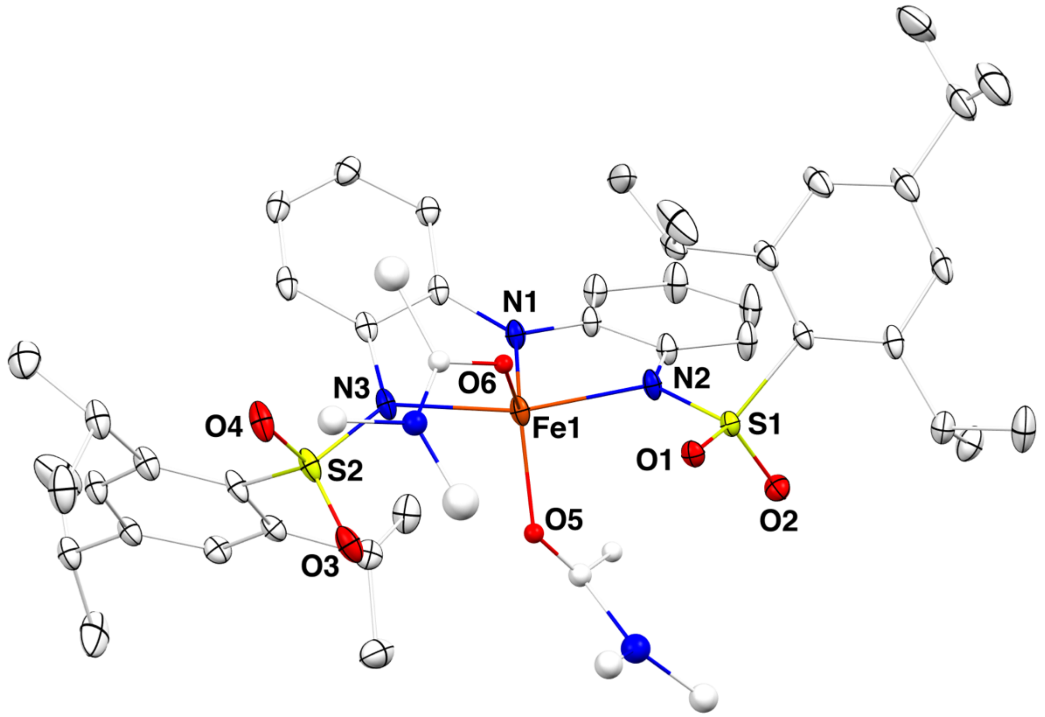

Figure S1. Thermal ellipsoid diagram of [Fe(ibaps)(DMA)$_2$]. The ellipsoids are drawn at the 30% probability level, and interstitial solvent molecules and hydrogen atoms are omitted for clarity.

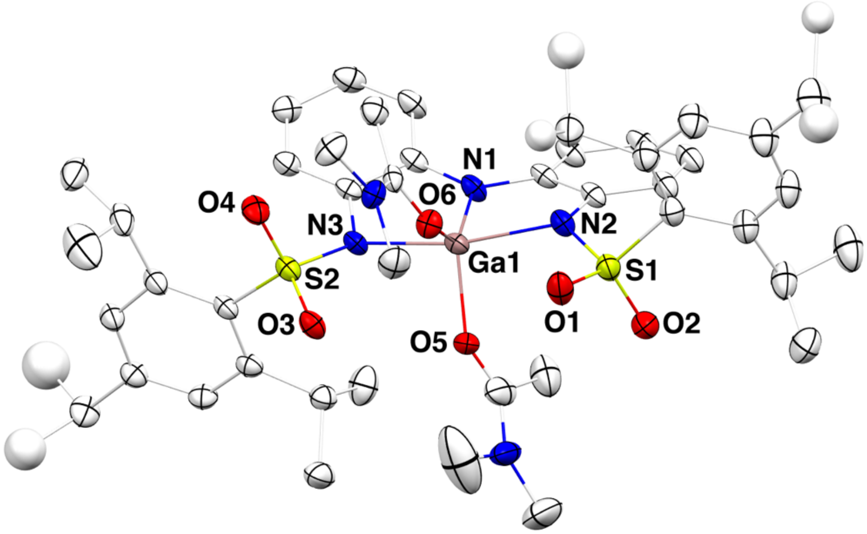

Figure S2. Thermal ellipsoid diagram of [Ga(ibaps)(DMA)$_2$]. The ellipsoids are drawn at the 30% probability level, and hydrogen atoms are omitted for clarity.

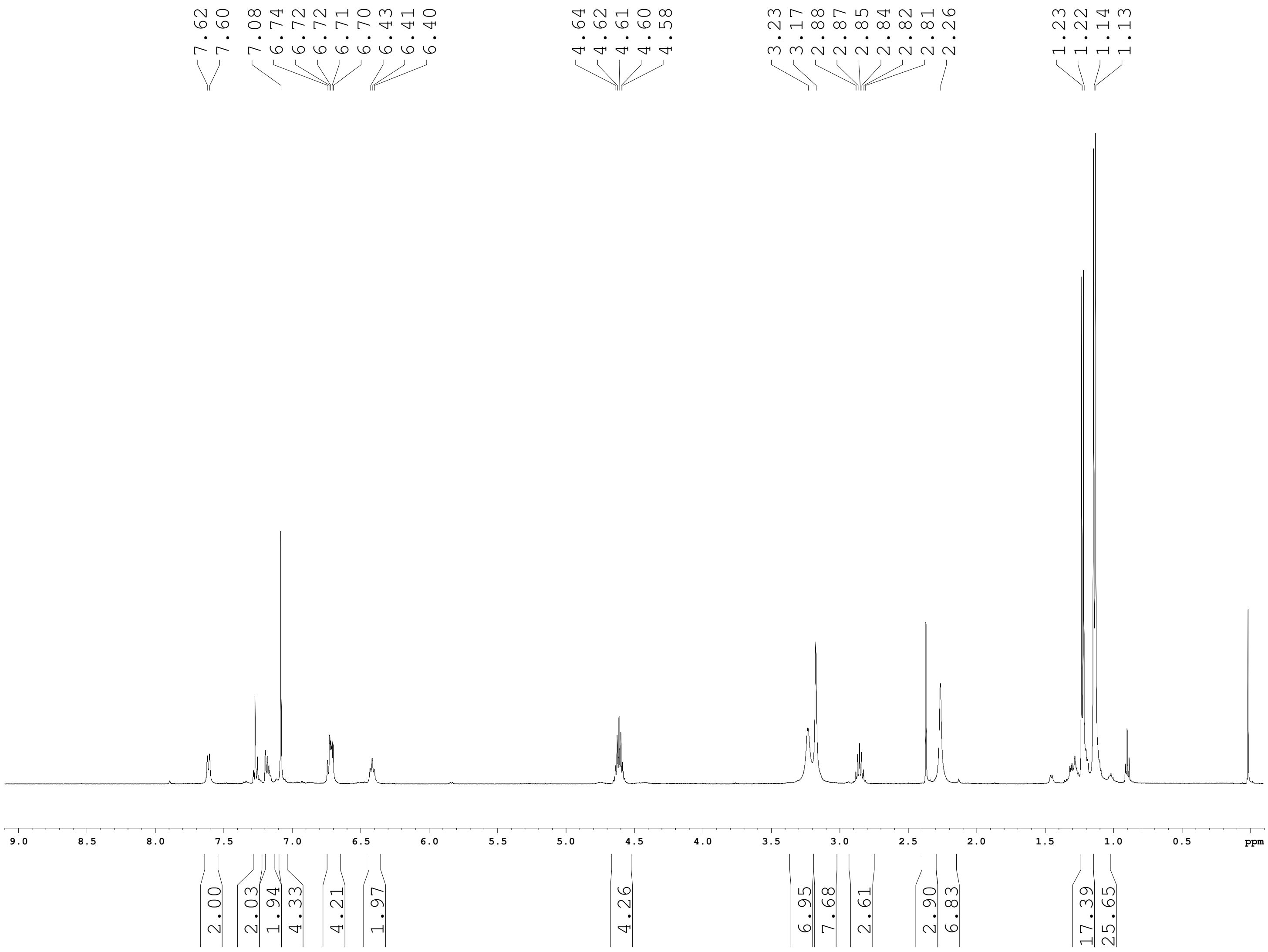

Figure S3. ^{1}H NMR spectrum of [Ga(ibaps)(DMA)$_2$] in $CDCl_3$.

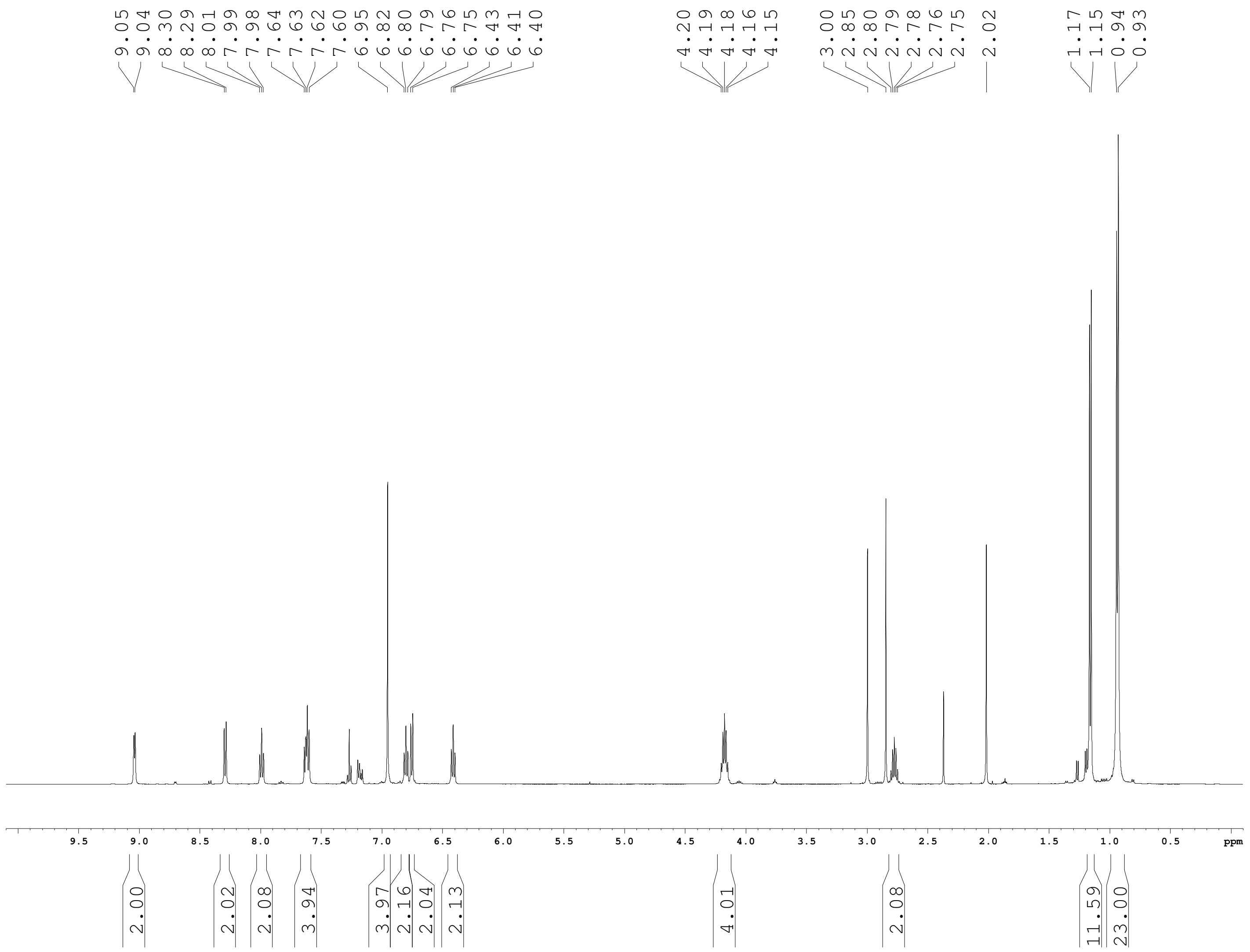

Figure S4. ^{1}H NMR spectrum of [Ga(ibaps)(bpy)] in $CDCl_3$.

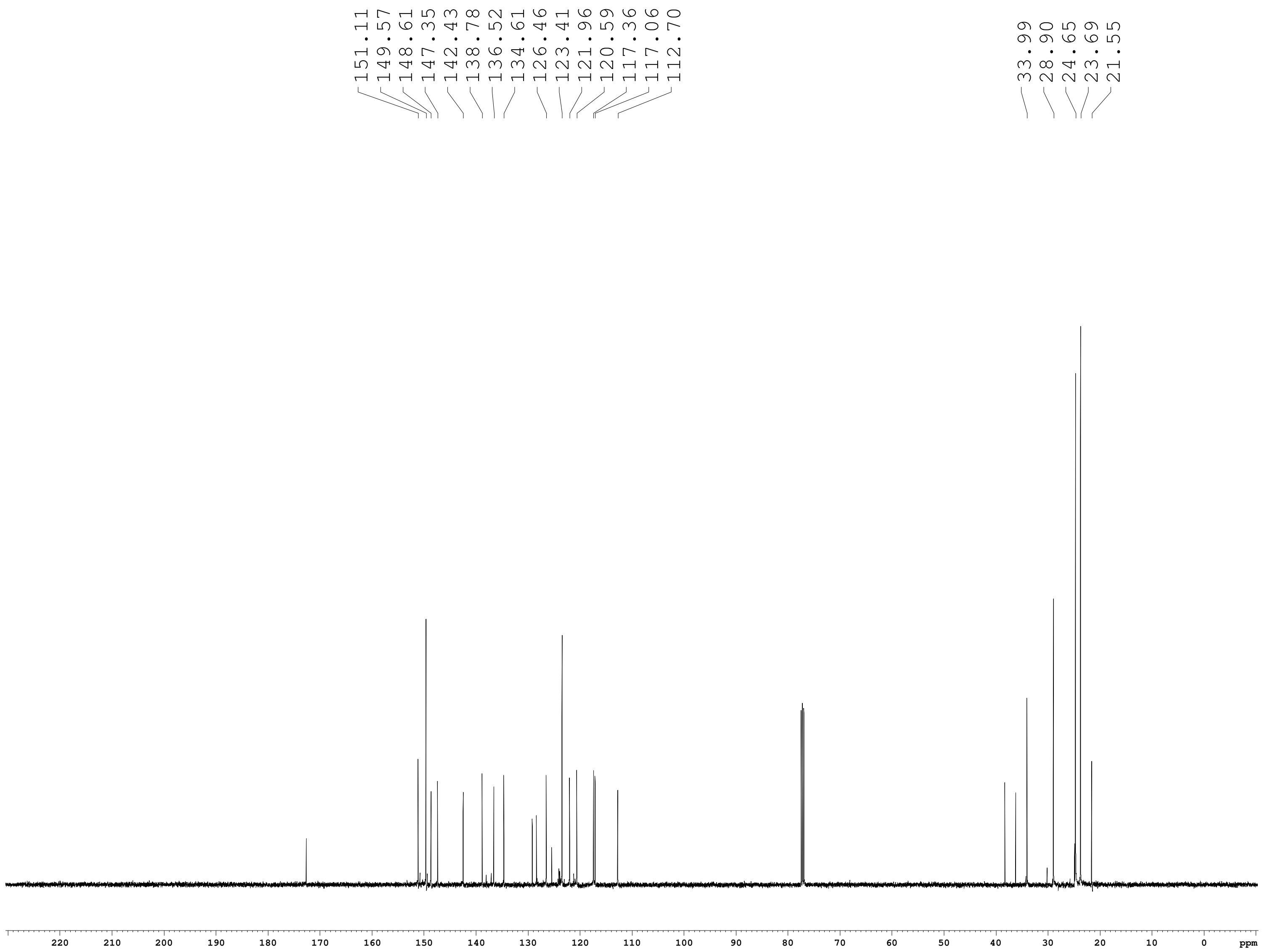

Figure S5. ^{13}C NMR spectrum of [Ga(ibaps)(bpy)] in $CDCl_3$.

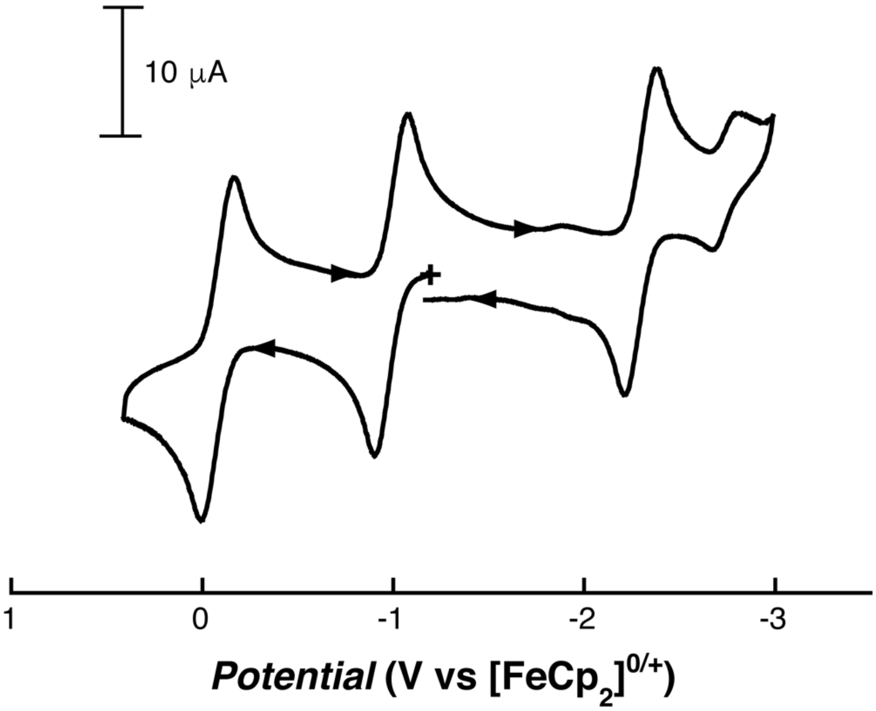

Figure S6. Cyclic voltammogram (small scan window) of **1** at room temperature in THF.

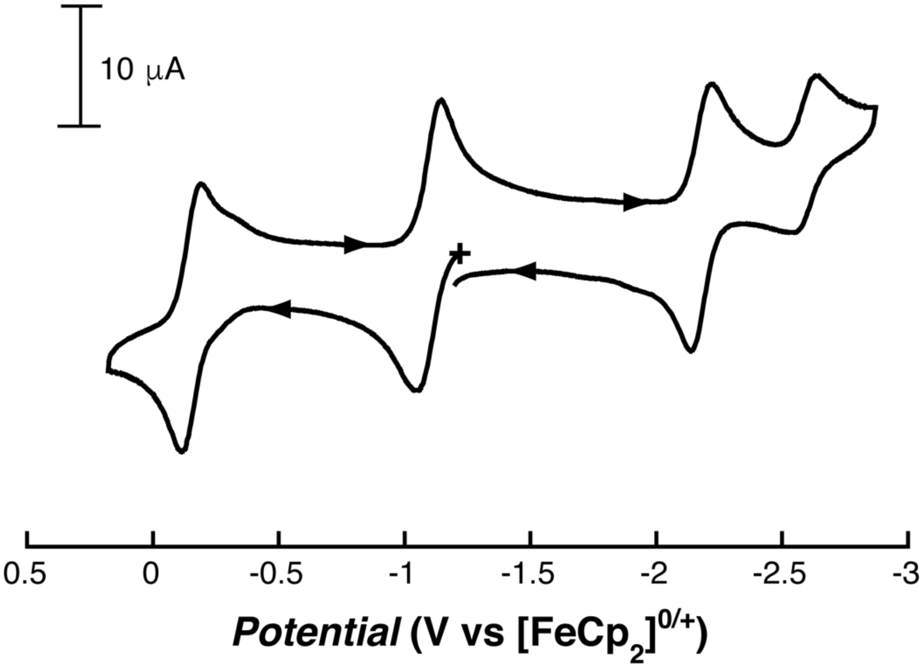

Figure S7. Cyclic voltammogram (small scan window) of **1** at room temperature in DMF.

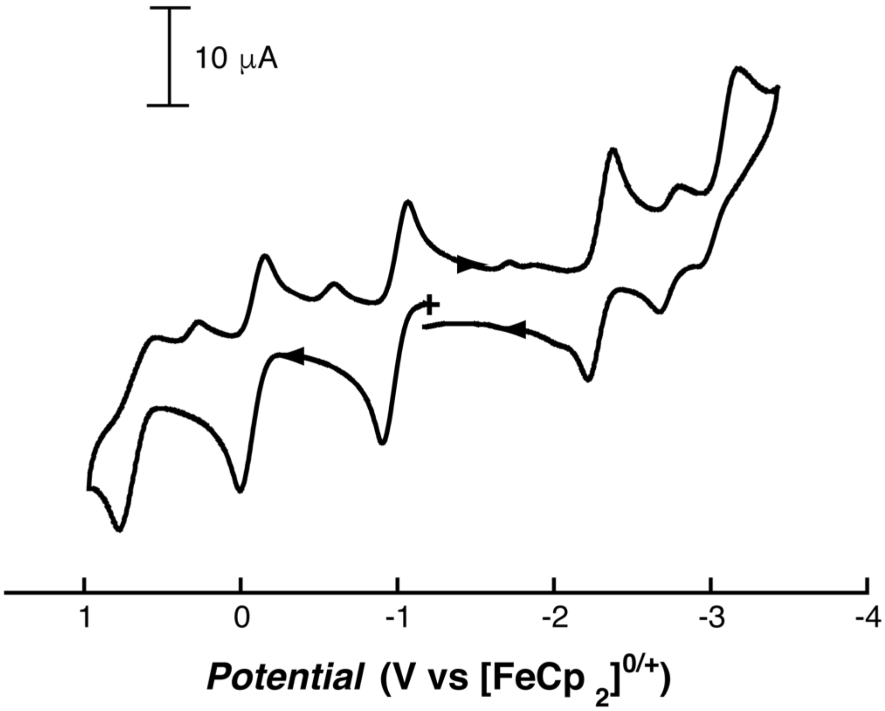

Figure S8. Cyclic voltammogram (full scan window) of **1** at room temperature in THF.

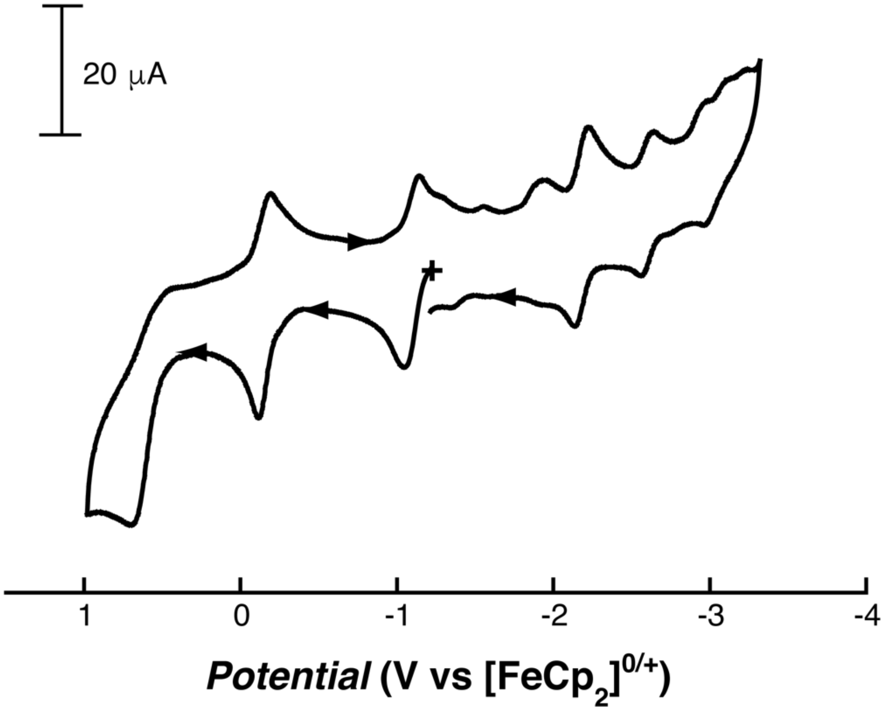

Figure S9. Cyclic voltammogram (full scan window) of **1** at room temperature in DMF.

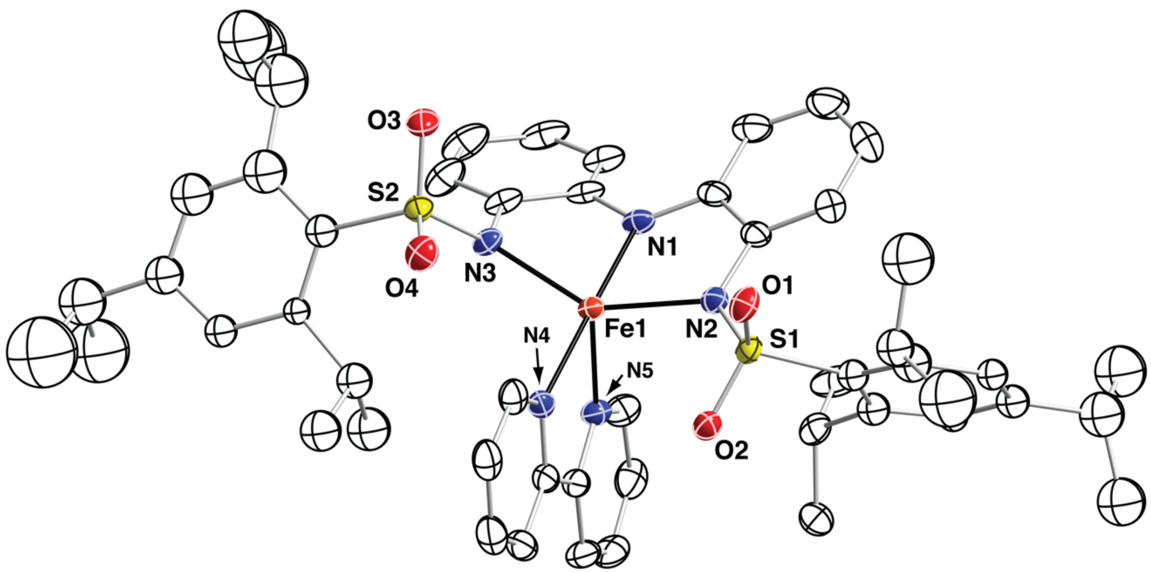

Figure S10. Thermal ellipsoid diagram of **1**. The ellipsoids are drawn at the 50% probability level, and hydrogen atoms are omitted for clarity.

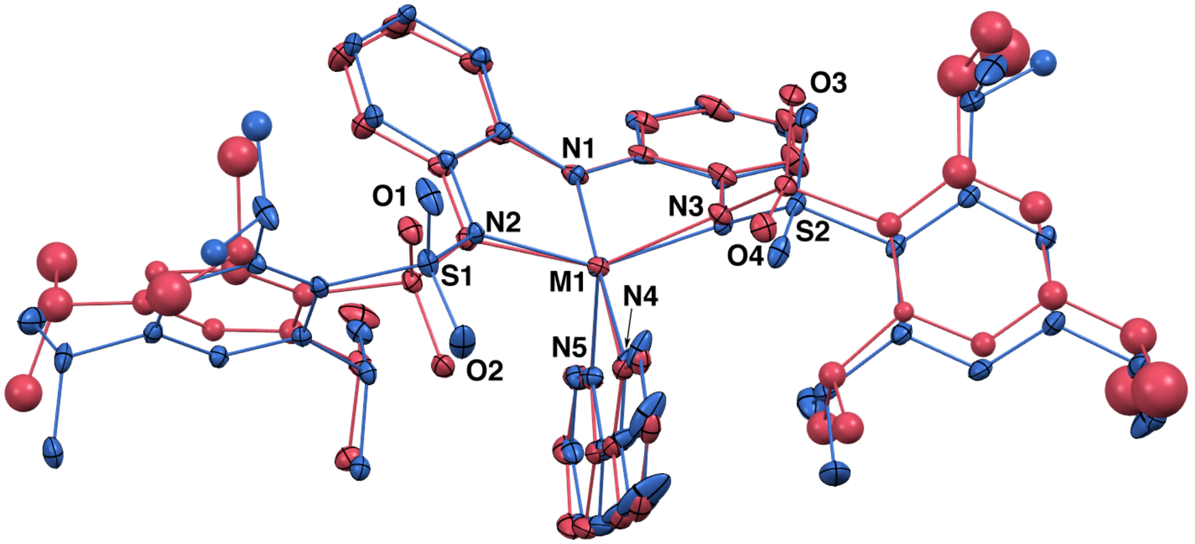

Figure S11. Overlay of the thermal ellipsoid diagrams of **1** (red) and **1-**Co (blue). The ellipsoids are drawn at the 30% probability level, and interstitial solvents, tetraethylammonium cations, and hydrogen atoms are omitted for clarity.

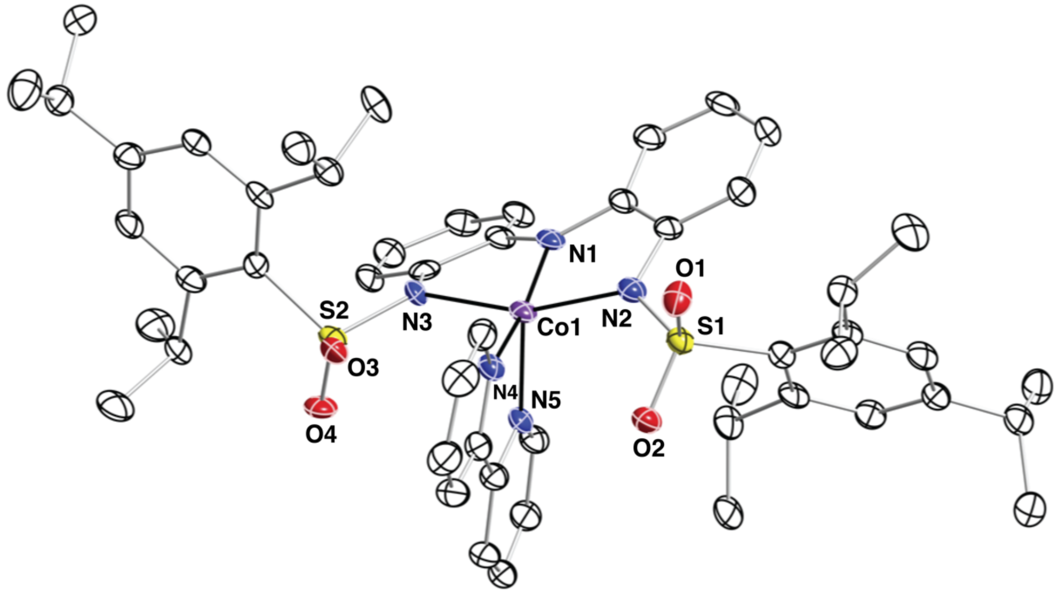

Figure S12. Thermal ellipsoid diagram of **2**-Co. The ellipsoids are drawn at the 50% probability level, and hydrogen atoms are omitted for clarity.

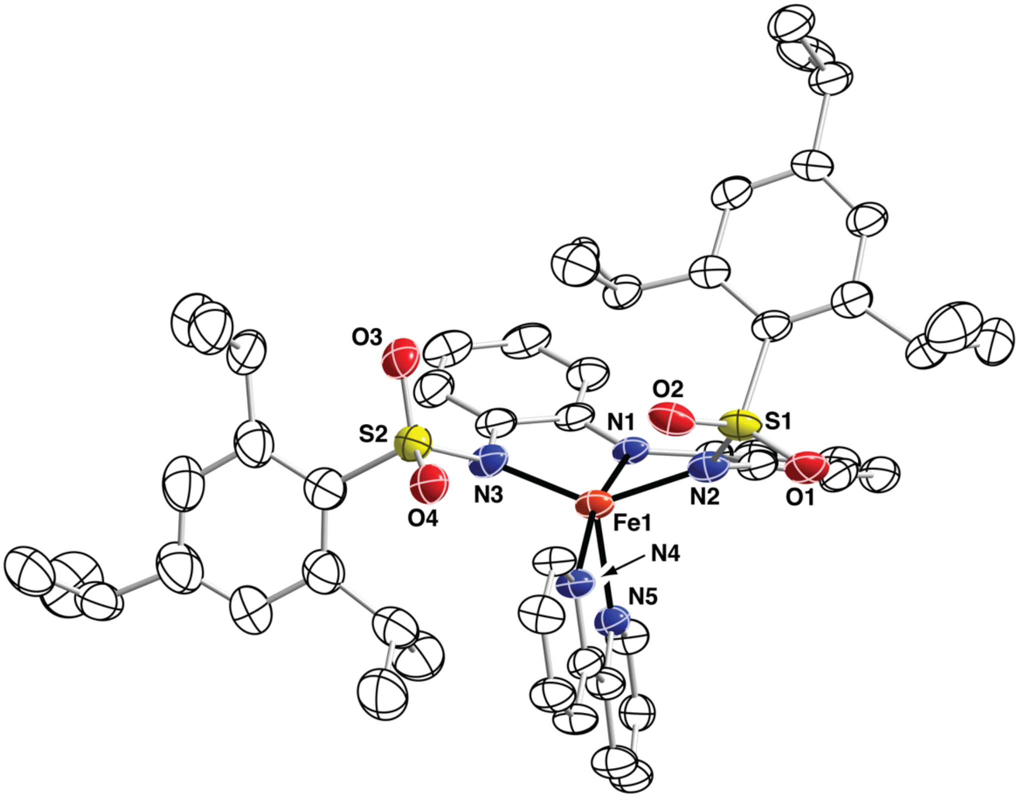

Figure S13. Thermal ellipsoid diagram of **2**. The ellipsoids are drawn at the 50% probability level, and hydrogen atoms are omitted for clarity.

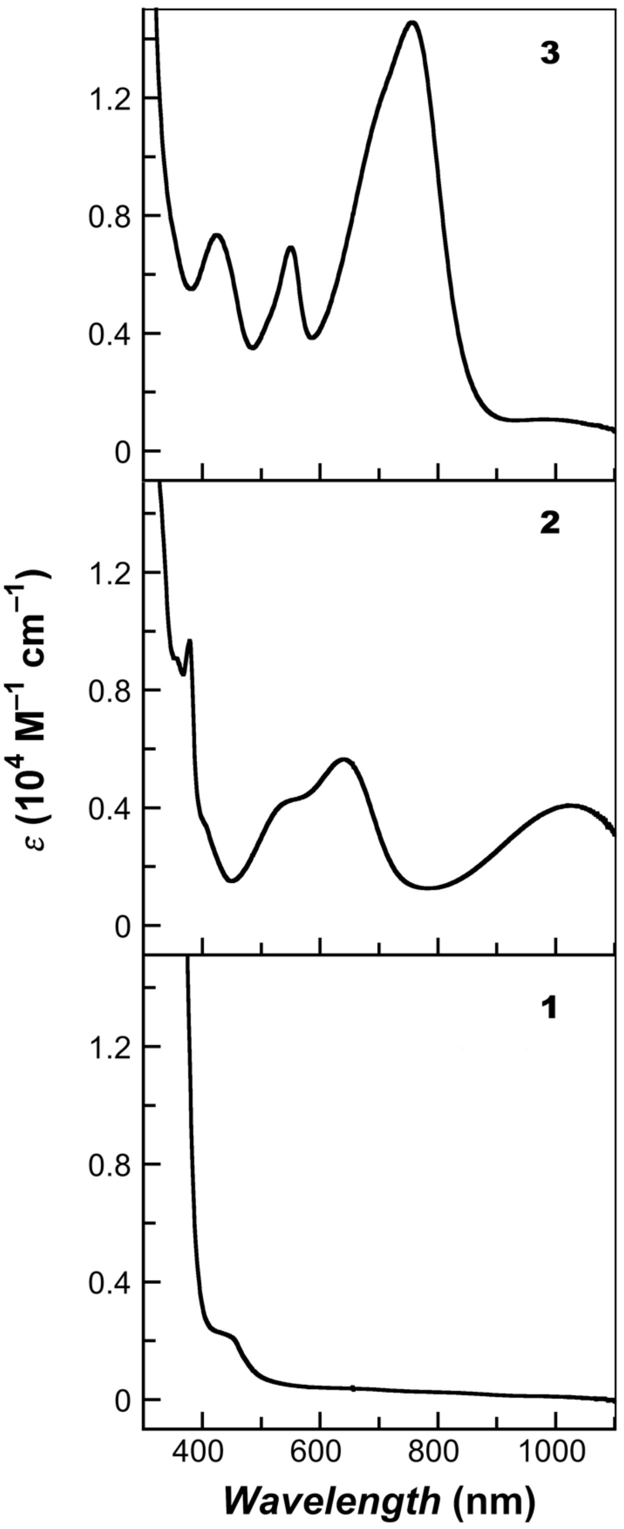

Figure S14. Electronic absorbance spectra of **1**-**3** recorded at room temperature in THF.

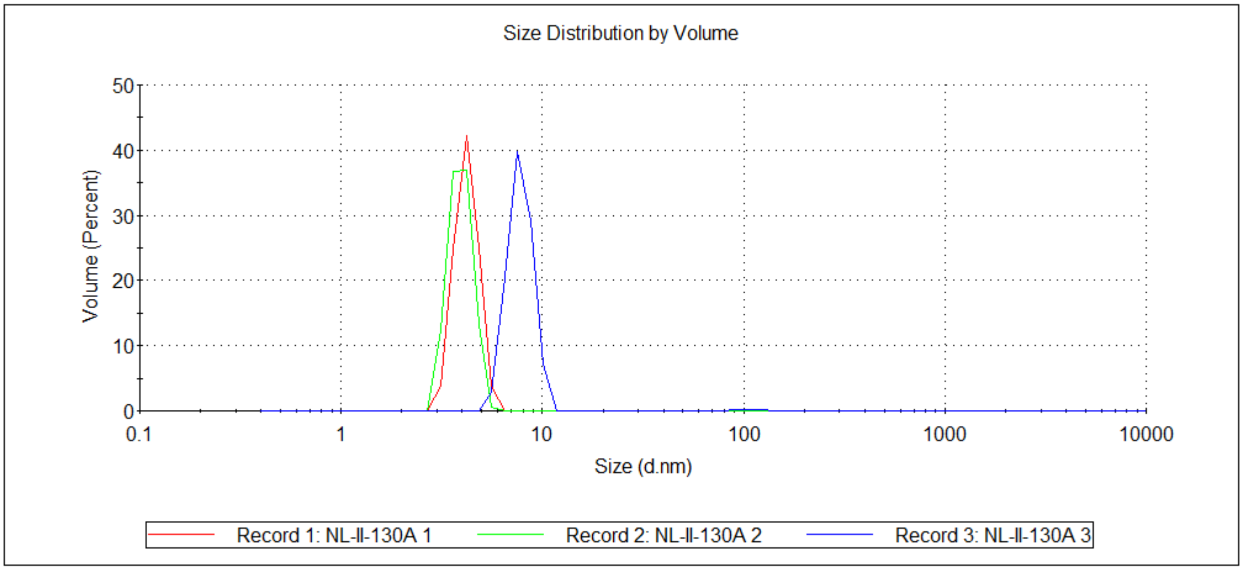

Figure S15. Size distribution by volume of the particles suspended in the reaction solutions was determined by three combined measurements composed of multiple scans, each of which were determined by backscattering method (DMF as solvent, 25 °C). The studies were done on mixtures of 1-azido-2-propylbenzene and **1** in the absence of molecular sieves that were allowed to react for 24 h at 115 °C in DMF. Experiments and measurements were conducted under Ar with no exposure to air. Average size distribution = 5.3 nm ± 2.2 nm (99.7%)

Table S1. Crystallographic data for **[Fe(ibaps)(DMA)$_2$]** and **[Ga(ibaps)(DMA)$_2$]**

Salt	**[Fe(ibaps)(DMA)$_2$]•1.5Hexane**	**[Ga(ibaps)(DMA)$_2$]**
CCDC#	1813040	1813043
Empirical Formula	$C_{59}H_{93}FeN_5O_6S_2$	$C_{50}H_{72}GaN_5O_6S_2$
fw	1088.35	972.96
T (K)	88(2)	133(2)
space group	*P*	$P2_1/c$
a (Å)	9.4615(9)	16.1329(11)
b (Å)	18.3994(18)	27.6083(19)
c (Å)	18.7650(19)	13.2048(9)
α (deg)	107.6055(12)	90
β (deg)	100.0237(12)	105.6127(8)
γ (deg)	93.8094(12)	90
Z	2	4
V (Å^3)	3041.4(5)	5664.4(7)
δ_{calcd} (Mg/m^3)	1.188	1.141
R1	0.0785	0.0624
wR2	0.2149	0.1769
GOF	1.032	1.051

wR2 = $[\Sigma[w(F_o^2 - F_c^2)^2] / \Sigma[w(F_o^2)^2]]^{1/2}$; R1 = $\Sigma||F_o|-|F_c|| / \Sigma|F_o|$; Goof = S = $[\Sigma[w(F_o^2 - F_c^2)^2] / (n-p)]^{1/2}$ where n is the number of reflections and p is the total number of parameters refined.

Table S2. Selected bond distances and angles for the [M(ibaps)(DMA)$_2$] complexes

Distances (Å) or angles (deg)	**[Fe(ibaps)(DMA)$_2$]**	**[Ga(ibaps)(DMA)$_2$]**
M1–N1	1.947(3)	1.881(2)
M1–N2	2.110(3)	2.068(2)
M1–N3	2.101(3)	2.048(3)
M1–O5	1.901(10)	1.899(2)
M1–O6	2.001(4)	1.889(3)
N1–C1	1.384(4)	1.387(4)
C1–C2	1.406(5)	1.395(4)
C2–C3	1.394(5)	1.375(5)
C3–C4	1.383(5)	1.368(5)
C4–C5	1.389(6)	1.391(5)
C5–C6	1.399(5)	1.375(5)
C1–C6	1.430(4)	1.422(4)
N3–C6	1.400(5)	1.417(4)
N1–C7	1.390(4)	1.394(4)
C7–C8	1.406(4)	1.395(4)
C8–C9	1.395(5)	1.390(5)
C9–C10	1.384(5)	1.374(6)
C10–C11	1.394(5)	1.386(5)
C11–C12	1.399(4)	1.388(4)
C7–C12	1.416(4)	1.412(5)
N2–C12	1.413(4)	1.421(4)
O5–M1–N1	125.7(3)	125.17(13)
O5–M1–O6	113.5(3)	100.23(18)
N1–M1–O6	120.79(18)	134.59(18)
O5–M1–N3	98.9(3)	96.39(10)
N1–M1–N3	78.22(11)	82.49(10)
O6–M1–N3	91.13(14)	94.30(12)
O5–M1–N2	94.4(3)	95.03(11)
N1–M1–N2	77.87(11)	81.96(11)
O6–M1–N2	101.72(14)	94.44(13)
N3–M1–N2	156.08(11)	164.16(10)
ϕ	36.79	28.09
τ_5	0.59	0.49

The dihedral angle, ϕ, is defined as the angle between the two planes formed by the two aryl rings of the [ibaps] backbone.

Table S3. Crystallographic data for **1-Co**, **2-Co**, **1**, and **2**

Salt	**1-Co•0.5Et$_2$O**	**2-Co•THF**	**1**	**2**
CCDC#	1813044	1813041	1813042	1813039
Empirical Formula	$C_{62}H_{87}CoN_6O_{4.5}S_2$	$C_{56}H_{70}CoN_5O_5S_2$	$C_{60}H_{82}FeN_6O_4S_2$	$C_{52}H_{62}FeN_5O_4S_2$
fw	1111.42	1016.22	1071.28	941.03
T (K)	88(2)	88(2)	88(2)	100.0
space group	$P2_1/n$	P	P	P
a (Å)	17.4061(12)	18.006(2)	10.6709(11)	10.175(2)
b (Å)	10.7648(8)	18.271(2)	17.4933(18)	16.218(3)
c (Å)	31.052(2)	18.568(2)	17.8715(18)	16.588(3)
α *(deg)*	90	110.9719(12)	113.4776(12)	84.402(6)
β *(deg)*	91.3044(9)	109.6022(13)	98.8759(12)	81.108(7)
γ *(deg)*	90	95.4770(13)	91.3574(13)	76.300(7)
Z	4	4	2	2
V (Å^3)	5816.7(7)	5209.7(11)	3009.9(5)	2622.3(10)
δ_{calcd} (Mg/m^3)	1.269	1.296	1.182	1.192
R1	0.0718	0.0790	0.0895	0.0961
wR2	0.1912	0.2105	0.2428	0.2401
GOF	1.046	1.009	1.036	1.041

wR2 = $[\Sigma[w(F_o^2 - F_c^2)^2] / \Sigma[w(F_o^2)^2]]^{1/2}$; R1 = $\Sigma||F_o| - |F_c|| / \Sigma|F_o|$; Goof = S = $[\Sigma[w(F_o^2 - F_c^2)^2] / (n-p)]^{1/2}$ where n is the number of reflections and p is the total number of parameters refined.

Table S4. Selected bond distances and angles for the [M(ibaps)bpy]^n complexes

Distances (Å) or angles (deg)	**1-Co**	**2-Co**	**1**	**2**
M1–N1	1.970(3)	1.841(3)	2.032(3)	1.992(5)
M1–N2	2.113(3)	1.974(3)	2.166(3)	2.066(6)
M1–N3	2.159(3)	1.989(3)	2.150(3)	2.064(6)
M1–N4	2.132(3)	1.942(3)	2.197(3)	2.157(5)
M1–N5	2.077(3)	1.977(3)	2.143(3)	2.132(5)
N1–C1	1.389(4)	1.393(5)	1.390(5)	1.377(8)
C1–C2	1.404(4)	1.397(5)	1.407(5)	1.393(9)
C2–C3	1.394(5)	1.392(5)	1.387(6)	1.371(10)
C3–C4	1.383(5)	1.376(5)	1.374(7)	1.404(11)
C4–C5	1.395(5)	1.384(5)	1.394(6)	1.357(10)
C5–C6	1.394(5)	1.406(5)	1.388(6)	1.399(10)
C1–C6	1.429(4)	1.430(5)	1.419(6)	1.404(9)
N3–C6	1.426(4)	1.395(5)	1.430(4)	1.408(9)
N1–C7	1.372(4)	1.393(5)	1.373(5)	1.399(8)
C7–C8	1.409(4)	1.404(5)	1.409(5)	1.384(9)
C8–C9	1.395(5)	1.383(5)	1.384(7)	1.386(10)
C9–C10	1.382(5)	1.387(6)	1.382(8)	1.394(11)
C10–C11	1.393(5)	1.392(6)	1.394(6)	1.389(10)
C11–C12	1.398(5)	1.406(5)	1.394(6)	1.367(10)
C7–C12	1.439(4)	1.413(5)	1.433(5)	1.434(10)
N2–C12	1.421(4)	1.409(5)	1.418(5)	1.433(8)
N1–M1–N5	106.09(11)	111.02(13)	103.34(12)	92.9(2)
N1–M1–N3	78.92(11)	81.72(13)	77.76(12)	78.2(2)
N5–M1–N3	102.02(11)	89.39(12)	107.37(12)	107.4(2)
N1–M1–N2	79.23(11)	82.10(13)	76.15(12)	78.3(2)
N5–M1–N2	108.20(11)	108.01(13)	101.10(12)	104.5(2)
N3–M1–N2	146.54(11)	159.60(13)	144.98(12)	140.9(2)
N1–M1–N4	177.15(12)	167.45(13)	178.72(12)	166.8(2)
N5–M1–N4	76.49(12)	81.50(13)	75.38(12)	76.0(2)
N3–M1–N4	99.44(11)	97.99(13)	102.69(11)	111.7(2)
N2–M1–N4	101.22(11)	95.12(13)	103.91(11)	97.4(2)
ϕ	33.75	28.45	33.28	27.29
τ_5	0.51	0.13	0.56	0.43

Table S5. Selected bond distances and angles for the second molecule of **2-Co**

Distances (Å) or angles (deg)	**2-Co**
Co2–N6	1.835(3)
Co2–N7	1.995(3)
Co2–N8	1.980(3)
Co2–N9	1.971(3)
Co2–N10	1.999(3)
N6–C53	1.396(5)
C53–C54	1.404(5)
C54–C55	1.389(5)
C55–C56	1.391(6)
C56–C57	1.374(6)
C57–C58	1.422(5)
C53–C58	1.420(5)
N8–C58	1.387(5)
N6–C59	1.405(5)
C59–C60	1.405(5)
C60–C61	1.391(6)
C61–C62	1.384(6)
C62–C63	1.381(6)
C63–C64	1.411(5)
C59–C64	1.410(5)
N7–C64	1.410(5)
N6–Co2–N10	126.69(14)
N6–Co2–N8	81.41(13)
N8–Co2–N10	89.39(13)
N6–Co2–N7	82.05(13)
N7–Co2–N10	104.49(12)
N8–Co2–N7	162.74(13)
N6–Co2–N9	153.33(13)
N9–Co2–N10	79.98(13)
N9–Co2–N8	100.35(13)
N9–Co2–N7	92.25(12)
ϕ	28.44
τ_5	0.16

The dihedral angle, ϕ, is defined as the angle between the planes formed by the two aryl rings of the [ibaps] backbone.

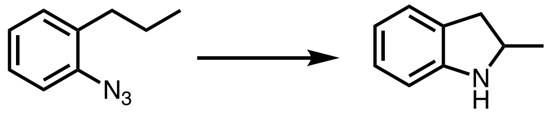

Table S6: Optimization and screening conditions for aliphatic C-H amination with **1.**

Entry	Catalyst	mol %	Solvent	T (ºC)	Yield Indoline [%]
1	**1**	10	Toluene:DMF 4:1	115	25
2	**1**	20	Toluene:DMF 4:1	115	33
3	**1**	50	Toluene:DMF 4:1	115	52
4	**1**	10	Toluene:DMF 4:1	rt	No reaction
5	**1**	10	Toluene:DMF 4:1	80	12
6	**1**	10	Toluene	115	34
7	**1**	10	DMF	115	57
8	**1**	10	DMA	115	27
9	**1**	10	CF_3-Toluene	115	17
10	Fe(OAc)/Bpy	10	DMF	115	No reaction
11	H_3ibaps	10	DMF	115	No reaction
12	___	___	DMF	115	No reaction

Reaction conditions: Drybox (Ar); in a pressure tube: catalyst (10 mol %), solvent (1 mL), 4Å MS, azide (1 equiv.), sealed tube taken out of the box and placed in a hot oil bath at a set temperature for heating, 24 h; analysis by GC-MS for product assignment, and GC-FID for yields. [a] mixture of 1:1 mol of $Fe(OAc)_2$ and bpy.

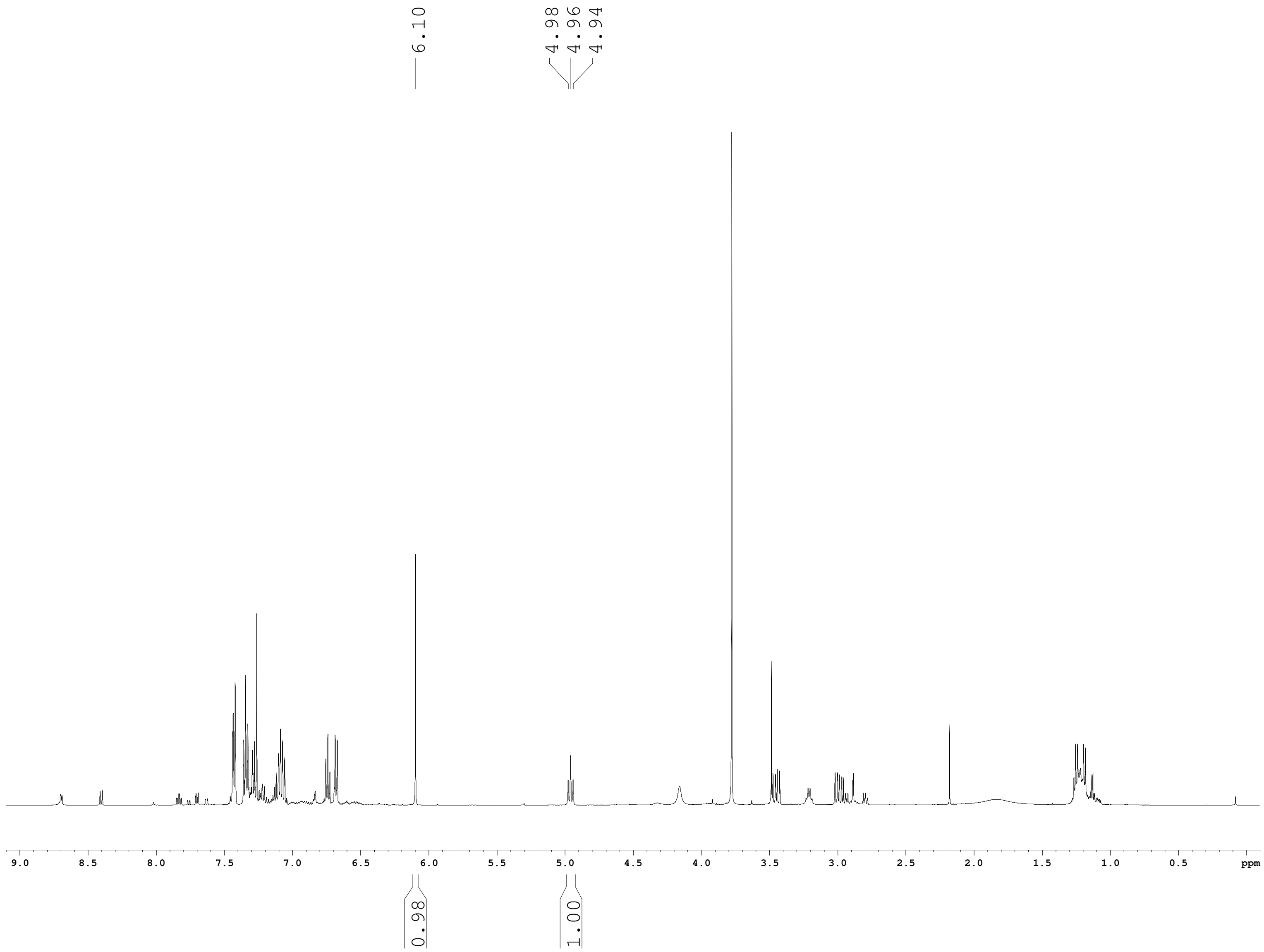

Figure S16. ^{1}H NMR spectrum of the product mixture from the reaction of 1-azido-2-phenethylbenzene with **1**.

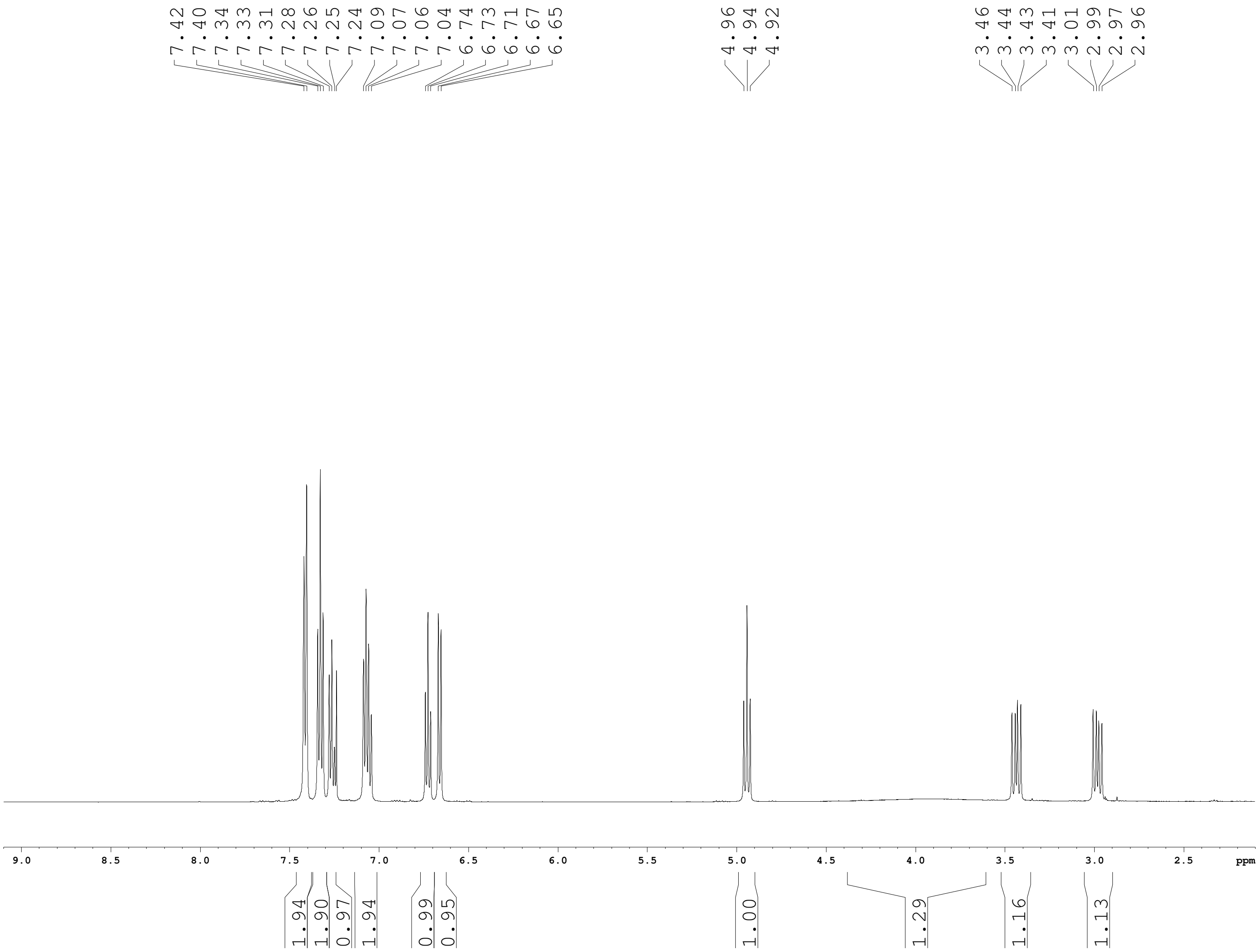

Figure S17. ^{1}H NMR spectrum of the isolated indoline product from the reaction of 1-azido-2-phenethylbenzene with **1**.

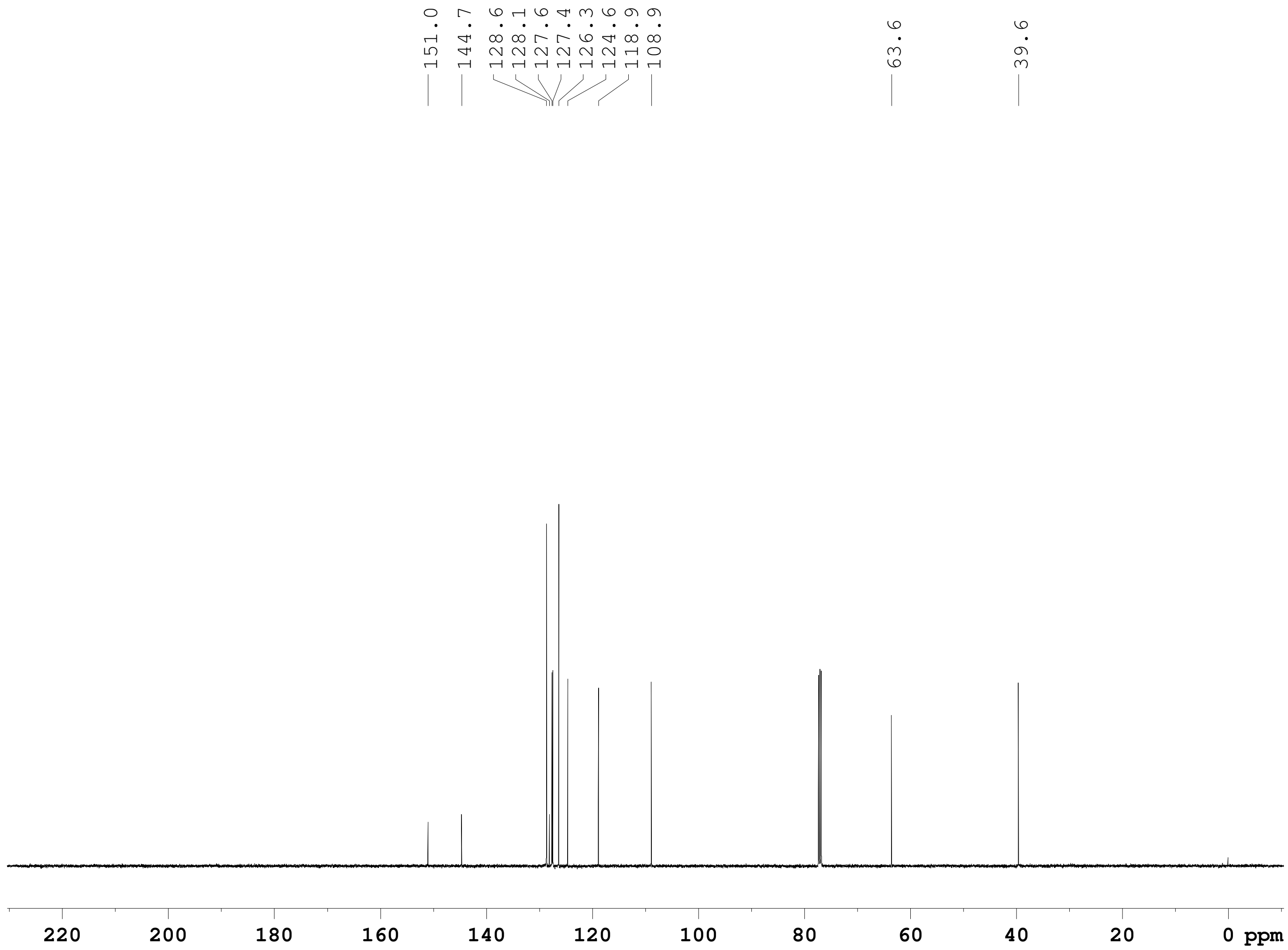

Figure S18. ^{13}C NMR spectrum of the isolated indoline product from the reaction of 1-azido-2-phenethylbenzene with **1**.

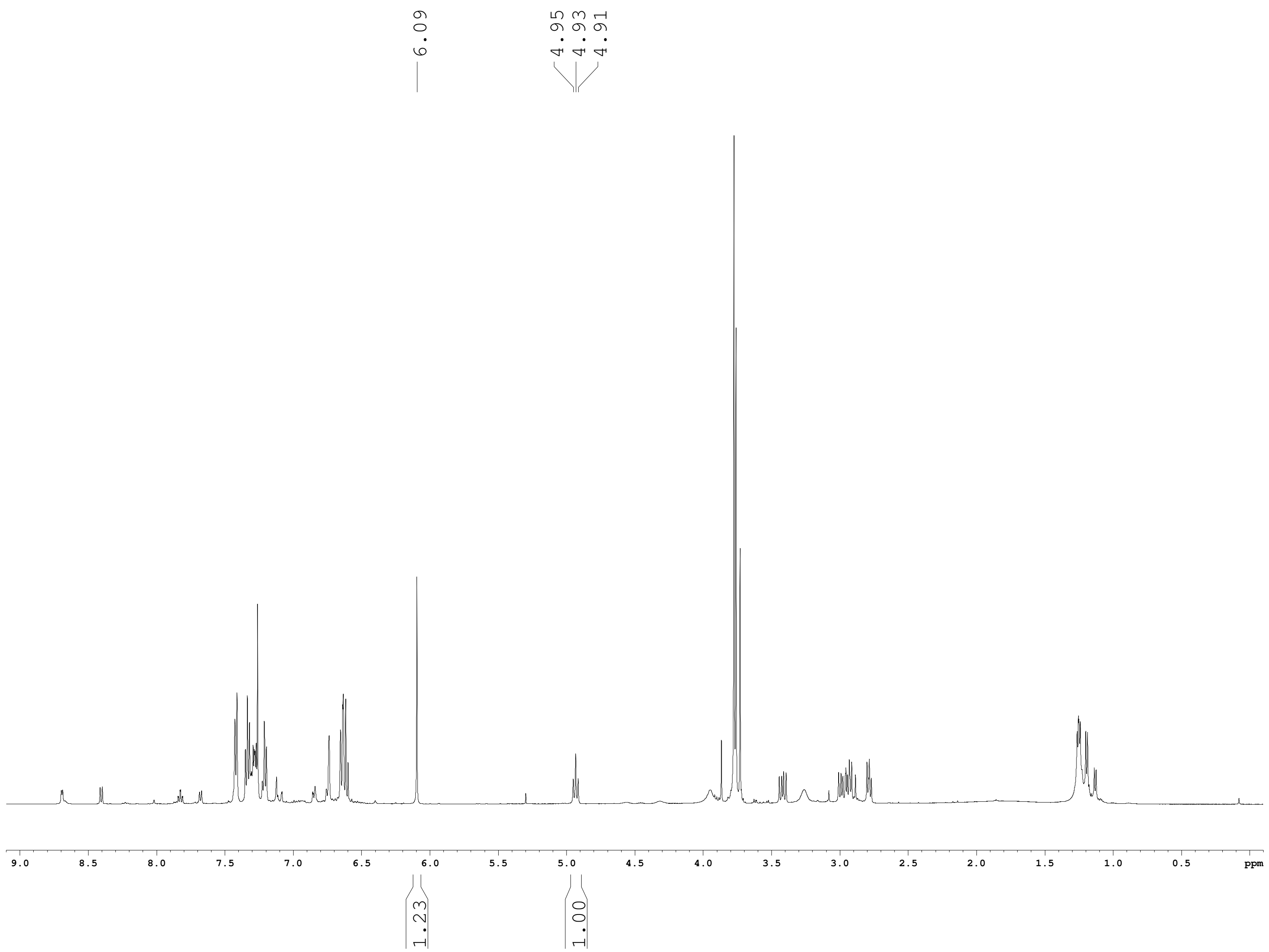

Figure S19. ^{1}H NMR spectrum of the product mixture from the reaction of 1-azido-4-methoxy-2-phenethylbenzene with **1**.

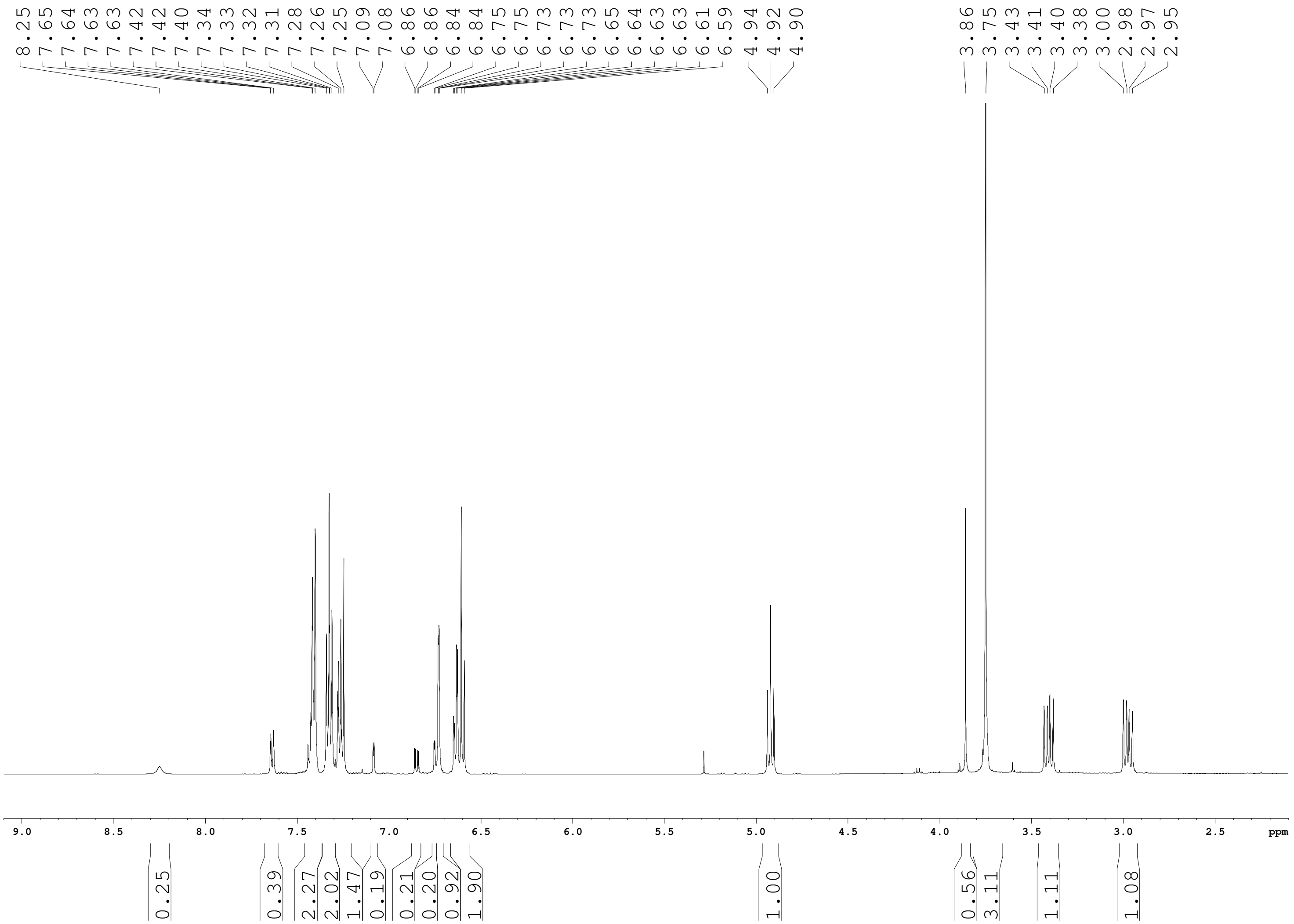

Figure S20. ^{1}H NMR spectrum of the isolated indoline and indole products from the reaction of 1-azido-4-methoxy-2-phenethylbenzene with **1**.

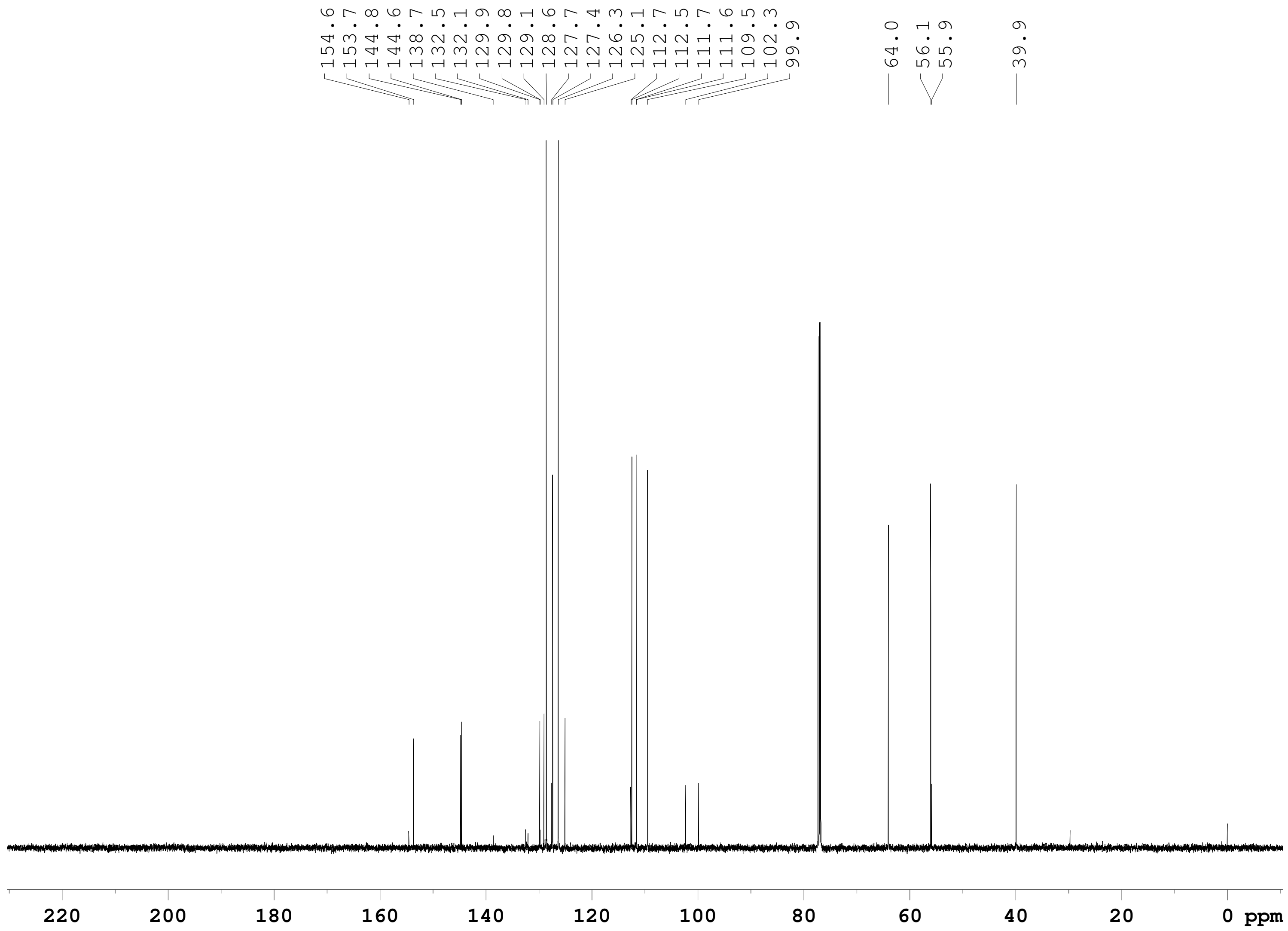

Figure S21. ^{13}C NMR spectrum of the isolated indoline and indole products from the reaction of 1-azido-4-methoxy-2-phenethylbenzene with **1**.

References.

(1) Sharma, S. K.; May, P. S.; Jones, M. B.; Lense, S.; Hardcastle, K. I.; MacBeth, C. E. Catalytic Dioxygen Activation by Co(ii) Complexes Employing a Coordinatively Versatile Ligand Scaffold. *Chem. Commun.* *47* (6), 1827–1829.

(2) Wilson, R. J.; Jones, J. R.; Bennett, M. V. Unprecedented Gallium-Nitrogen Anions: Synthesis and Characterization of [(Cl3Ga)3N]3- and [(Cl3Ga)2NSnMe3]2-. *Chem. Commun.* **2013**, *49* (44), 5049–5051.

(3) Sun, K.; Sachwani, R.; Richert, K. J.; Driver, T. G. Intramolecular Ir(I)-Catalyzed Benzylic C-H Bond Amination of Ortho-Substituted Aryl Azides. *Org. Lett.* **2009**, *11* (Copyright (C) 2011 American Chemical Society (ACS). All Rights Reserved.), 3598–3601.

(4) Connelly, N. G.; Geiger, W. E. Chemical Redox Agents for Organometallic Chemistry. *Chem. Rev.* **1996**, *96* (2), 877–910.

(5) Bruker AXS Inc, APEX2 Version 2014.11-0. Madison, WI 2014.

(6) Bruker AXS Inc, SAINT Version 8.34a. Madison, WI 2013.

(7) Sheldrick, G. M. SADABS. Bruker AXS, Inc: Madison 2014.

(8) Sheldrick, G. M. SHELXTL. Bruker AXS, Inc: Madison 2014.

(9) Wilson, A. J. C.; Geist, V. International Tables for Crystallography. Volume C: Mathematical, Physical and Chemical Tables. Kluwer Academic Publishers, Dordrecht/Boston/London 1992 (Published for the International Union of Crystallography), 883 Seiten, ISBN 0-792-3-16-38X. *Cryst. Res. Technol.* **1993**, *28* (1), 110–110.

(10) Spek, A. L. PLATON SQUEEZE: A Tool for the Calculation of the Disordered Solvent Contribution to the Calculated Structure Factors. *Acta Crystallogr. Sect. C Struct. Chem.* **2015**, *71* (1), 9–18.

(11) Spek, A. L. Structure Validation in Chemical Crystallography. *Acta Crystallogr. Sect. D Biol. Crystallogr.* **2009**, *65* (2), 148–155.
